# Ibrutinib modulates Aβ/tau pathology, neuroinflammation, and cognitive function in mouse models of Alzheimer's disease

**DOI:** 10.1111/acel.13332

**Published:** 2021-03-11

**Authors:** Hyun‐ju Lee, Seong Gak Jeon, Jieun Kim, Ri Jin Kang, Seong‐Min Kim, Kyung‐Min Han, HyunHee Park, Ki‐taek Kim, You Me Sung, Hye Yeon Nam, Young Ho Koh, Minseok Song, Kyoungho Suk, Hyang‐Sook Hoe

**Affiliations:** ^1^ Department of Neural Development and Disease Korea Brain Research Institute (KBRI) Daegu Korea; ^2^ Medical Device Development Center Daegu‐Gyeongbuk Medical Innovation Foundation (DGMIF) Daegu Korea; ^3^ Department of Life Sciences Yeungnam University Gyeongsan Korea; ^4^ Korea Mouse Phenotyping Center (KMPC) Seoul National University Seoul Korea; ^5^ Center for Biomedical Sciences Center for Infectious Diseases Division of Brain Disease Korea National Institute of Health Heungdeok‐gu Korea; ^6^ Department of Pharmacology Brain Science & Engineering Institute School of Medicine Kyungpook National University Daegu Korea; ^7^ Department of Brain and Cognitive Sciences Daegu Gyeongbuk Institute of Science & Technology Daegu Korea

**Keywords:** 5xFAD mice, Alzheimer's disease, amyloid beta, ibrutinib, neuroinflammation, PS19 mice, spinogenesis, tau

## Abstract

We previously demonstrated that ibrutinib modulates LPS‐induced neuroinflammation in vitro and in vivo, but its effects on the pathology of Alzheimer's disease (AD) and cognitive function have not been investigated. Here, we investigated the effects of ibrutinib in two mouse models of AD. In 5xFAD mice, ibrutinib injection significantly reduced Aβ plaque levels by promoting the non‐amyloidogenic pathway of APP cleavage, decreased Aβ‐induced neuroinflammatory responses, and significantly downregulated phosphorylation of tau by reducing levels of phosphorylated cyclin‐dependent kinase‐5 (p‐CDK5). Importantly, tau‐mediated neuroinflammation and tau phosphorylation were also alleviated by ibrutinib injection in PS19 mice. In 5xFAD mice, ibrutinib improved long‐term memory and dendritic spine number, whereas in PS19 mice, ibrutinib did not alter short‐ and long‐term memory but promoted dendritic spinogenesis. Interestingly, the induction of dendritic spinogenesis by ibrutinib was dependent on the phosphorylation of phosphoinositide 3‐kinase (PI3K). Overall, our results suggest that ibrutinib modulates AD‐associated pathology and cognitive function and may be a potential therapy for AD.

## Graphical abstract

This study evaluated the effects of ibrutinib on Aβ/tau pathology and cognitive function in two mouse models of Alzheimer's disease (AD). Ibrutinib reduced tau phosphorylation, neuroinflammation, or Aβ accumulation in 5xFAD mice and PS19 mice. Interestingly, ibrutinib injection improved long‐term memory and dendritic spine number in 5xFAD mice. Moreover, the induction of spinogenesis by ibrutinib was dependent on PI3K phosphorylation. These data suggest that ibrutinib holds potential as a therapeutic drug for AD.

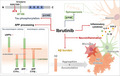

## 1 INTRODUCTION

Alzheimer's disease (AD) is a neurodegenerative disease that usually progresses slowly but gradually worsens after onset. Despite extensive study, the exact pathological mechanisms and appropriate therapeutic strategies for AD remain unclear. AD is characterized by two neuropathological symptoms: amyloid beta (Aβ) plaques, and neurofibrillary tangles (NFTs) (Haass & Selkoe, 2007). Aβ is generated by proteolytic processing of amyloid beta precursor protein (APP), and accumulation of Aβ impairs memory, reduces the formation of dendritic spines, and increases neuroinflammation and tauopathy (O'Brien & Wong, 2011). In its normal monomeric form, the protein tau plays an important role in stabilizing microtubules (Mietelska‐Porowska et al., 2014). However, under pathological conditions, hyperphosphorylated tau detaches from microtubules, is ubiquitinated, and aggregates into paired helical filaments (PHFs) (Lasagna‐Reeves et al., 2012). PHFs form neurotoxic and synaptic NFTs, which lead to dendritic spine loss, cognitive dysfunction, and finally progression to neurodegenerative disease (Gao et al., 2018). Although the molecular mechanisms by which Aβ and NFTs affect synaptic and cognitive function have yet to be fully elucidated (Nam et al., 2019), drugs that inhibit and/or prevent the accumulation of Aβ and hyperphosphorylation of tau may be useful for target‐based treatment of AD.

Ibrutinib is an FDA‐approved small molecule for treating B‐cell lymphoma, including chronic lymphocytic leukemia and mantle cell lymphoma (Burger et al., 2017). Ibrutinib binds irreversibly to a cysteine residue in the ATP‐binding site of Bruton's tyrosine kinase (BTK) to inhibit the activity of the enzyme (Campbell et al., 2018). BTK is involved in the phosphorylation of Toll‐like receptors and signaling cascades that produce proinflammatory cytokines (Lee et al., 2012), and thus, inhibiting BTK is expected to reduce inflammation. Accordingly, ibrutinib modulates immune cell activation and reduces proinflammatory cytokine levels in a pulmonary inflammation model (de Porto et al., 2019), and we and others have reported that ibrutinib crosses the blood–brain barrier (BBB) to alleviate LPS‐evoked neuroinflammation and ischemic stroke‐induced NLRP3 inflammasome activation (Ito et al., 2015; Mason et al., 2017; Nam et al., 2018).

However, the potential of ibrutinib to modulate the pathology of neurodegenerative diseases such as AD has not been fully addressed. BTK expression is upregulated postmortem in the brains of AD patients and 5xFAD mice (mouse model of AD) (Keaney et al., 2019), and inhibition of BTK suppresses microglial activation and synaptic loss by deactivating PLCγ2 (Keaney et al., 2019). Given that reactive microglia are critical in the pathogenesis of AD, these observations imply that suppression of BTK might be a potential target for regulating AD pathology via inhibition of microglial activation.

Interestingly, ibrutinib irreversibly binds to cysteine residues of kinases other than BTK, including EGFR family kinases, BLK, and JAK3 besides BTK (TEC family kinases) (Berglof et al., 2015), suggesting that ibrutinib might have therapeutic effects on non‐B‐cell cancer. Importantly, cancer patients treated with chemotherapy exhibit decreased risk of AD (Musicco et al., 2013), and an anticancer inhibitor of EGFR improves cognitive function in AD model mice (Wang et al., 2012). Taken together, these previous observations led us to hypothesize that the anticancer drug ibrutinib may modulate AD symptoms.

To test this hypothesis, here we examined the effect of ibrutinib on AD pathology. In 5xFAD mice (a model of AD in which Aβ is overexpressed), ibrutinib suppressed Aβ and tau pathology and neuroinflammation, and in PS19 mice (a tauopathy model), ibrutinib significantly alleviated tau phosphorylation and tau‐evoked neuroinflammation. Moreover, ibrutinib improved long‐term memory of 5xFAD mice by enhancing the formation of dendritic spines. Interestingly, both dendritic spine number and PI3K phosphorylation were significantly increased in ibrutinib‐injected PS19 mice. In addition, increased dendritic spine number in ibrutinib‐treated primary hippocampal neurons was dependent on PI3K. Overall, the results of this study suggest that ibrutinib can modulate synaptic/cognitive function, neuroinflammatory responses, and AD pathology.

## 2 RESULTS

### 2.1 Ibrutinib decreases Aβ plaque accumulation in 5xFAD mice

Ibrutinib can cross the BBB (Bernard et al., 2015; Mason et al., 2017), and here, we further examined the distribution of ibrutinib in the brains of wild‐type (WT) mice after intraperitoneal administration of ibrutinib (10 mg/kg; i.p. daily for 14 consecutive days). Analysis by high‐performance liquid chromatography demonstrated that the ibrutinib concentration in the brain was significantly higher in ibrutinib‐injected mice (122.008 ng of ibrutinib/g of brain tissue) than in vehicle‐treated mice (15.165 ng of ibrutinib/g of brain tissue), confirming that ibrutinib penetrates the BBB in WT mice (Table S1).

**FIGURE 1:**
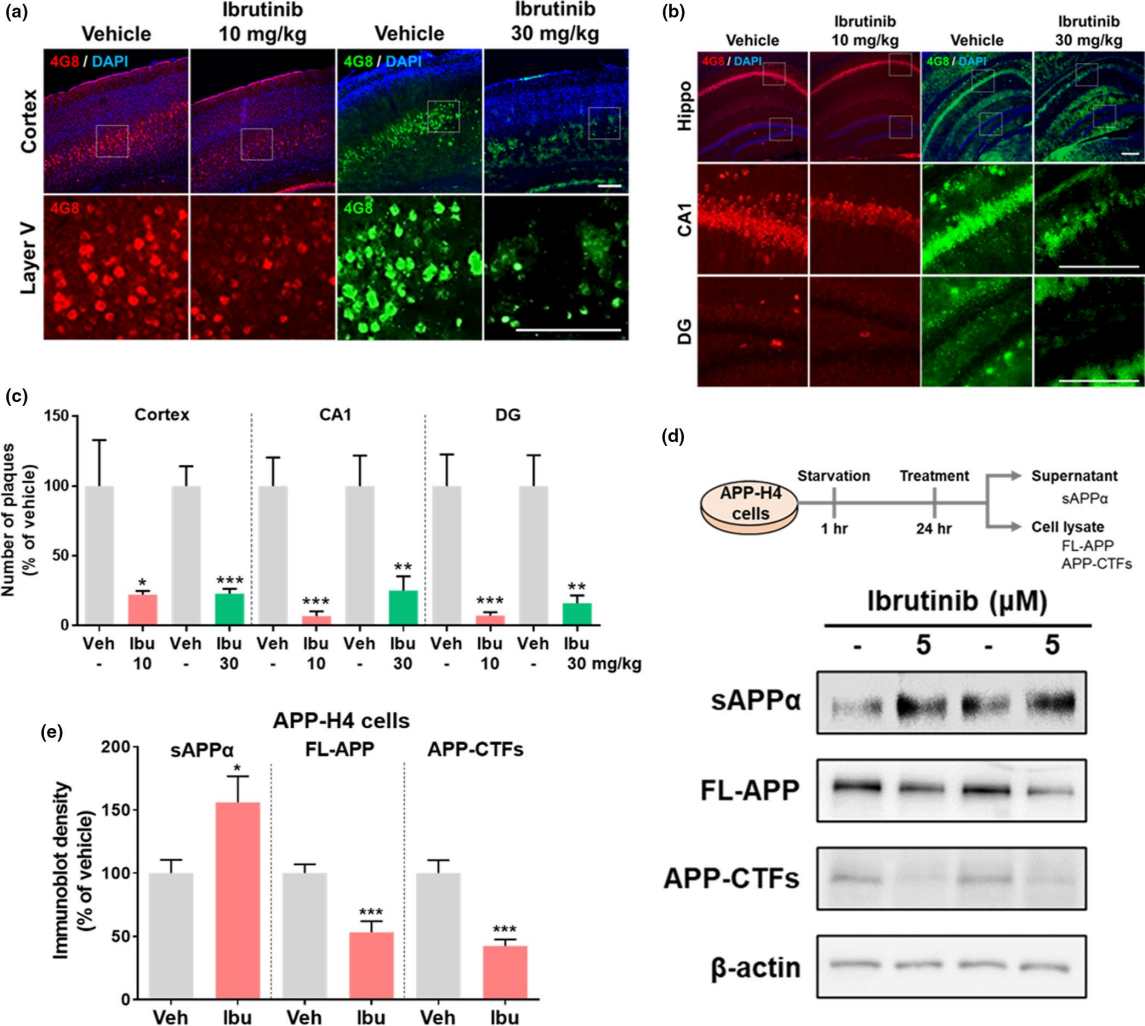
Ibrutinib reduces Aβ plaque burden in 3‐month‐old 5xFAD mice. (a and b) Ibrutinib or vehicle was injected (i.p.) daily for 14 consecutive days, and brain sections were ‐immunostained with an anti‐4G8 antibody. (c) Quantification of data from a and b (*n* = 3–4 mice/group). (d) H4 cells overexpressing APP (APP‐H4) were exposed to ibrutinib or vehicle (1% DMSO) for 3 hr and immunoblotted with anti‐sAPPα and anti‐c1/6.1 antibodies. (e) Quantification of data from d (*n* = 12 /group). Scale bar = 100 μm. Data are presented as the mean ± *SEM* (*p < 0.05, **p < 0.01, and ***p < 0.001 vs. vehicle)

We then examined whether ibrutinib alters the number of Aβ plaques in the brains of 5xFAD mice. We selected a dose of 10 mg/kg for injection (i.p.) based on its ability to efficiently reduce the neuroinflammatory response in LPS‐treated wild‐type mice (Nam et al., 2018). Three‐month‐old 5xFAD mice administered vehicle (5% DMSO +30% PEG +5% Tween‐80; i.p.) or ibrutinib (10 or 30 mg/kg; i.p.) by injection daily for 14 consecutive days, followed by immunostaining of brain sections with an anti‐4G8 antibody. Aβ plaque numbers in the cortex, CA1, and DG (dentate gyrus) of 3‐month‐old 5xFAD mice were significantly reduced by ibrutinib injection (Figure 1a–c). Orally administered ibrutinib (30 mg/kg; p.o. daily for 30 days) also significantly decreased Aβ plaque levels in the cortex and DG of 3‐month‐old 5xFAD mice (Figure S1a–c). Similar studies in older mice revealed that ibrutinib (10 mg/kg; i.p. daily for 14 days) significantly reduced Aβ plaque loads in the cortex and hippocampus CA1 of 6‐month‐old but not 12‐month‐old 5xFAD mice (Figure S1a–c). These data indicate that ibrutinib modulates Aβ plaque formation in the early and moderate stages of Aβ pathology. Consistent with our previous study, an ibrutinib dose of 10 mg/kg (i.p., daily for 14 days) efficiently reduced Aβ pathology in 3‐month‐old 5xFAD mice, and therefore, this dose and 3‐month‐old 5xFAD and PS19 mice were used in all subsequent experiments.

Aβ plaques are produced via aggregation of APP cleaved by β‐secretase (Zhang et al., 2011). To probe the mechanism by which ibrutinib reduces Aβ plaque numbers, H4 cells overexpressing APP were exposed to ibrutinib (5 μM) or vehicle (1% DMSO) for 24 hr and immunoblotted with anti‐C1/6.1 antibodies (recognizing APP‐C‐terminal fragments (APP‐CTFs) and full‐length APP (FL‐APP)) and anti‐secreted APPα (sAPPα) antibodies (Table S2). Ibrutinib significantly increased sAPPα levels in the conditioned medium and decreased APP‐CTF and FL‐APP levels in cell lysates (Figure 1d,e), suggesting that ibrutinib reduces Aβ plaque burden by promoting the non‐amyloidogenic pathway of APP cleavage.

### 2.2 Ibrutinib reduces Aβ plaque‐associated gliosis and proinflammatory cytokine levels in 5xFAD mice

**FIGURE 2:**
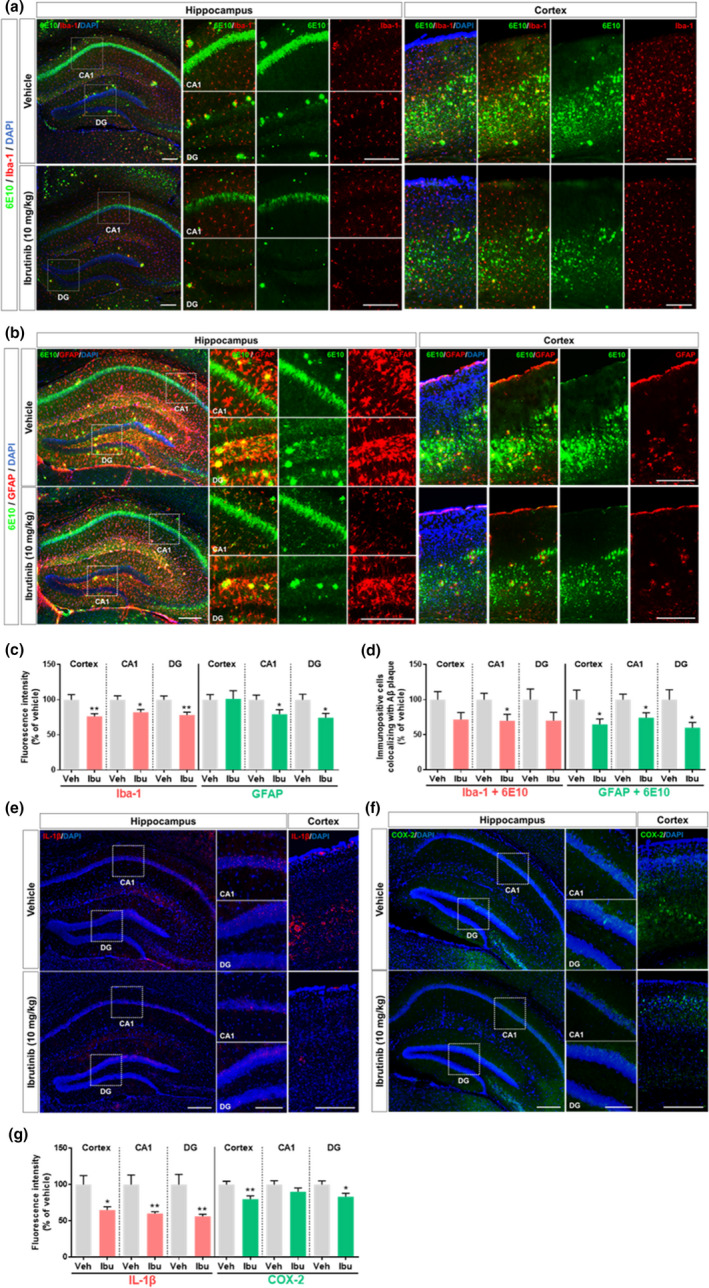
Ibrutinib suppresses Aβ‐mediated gliosis and levels of the proinflammatory cytokines IL‐1β and COX‐2 in 3‐month‐old 5xFAD mice. (a and b) Ibrutinib or vehicle was injected (i.p.) daily for 14 consecutive days, and brain sections were immunostained with anti‐Iba‐1, anti‐GFAP, and 6E10 antibodies. (c and d) Quantification of data from a and b (*n* = 4 mice/group). (e and f) Ibrutinib or vehicle was injected (i.p.) daily for 14 consecutive days, and brain sections were immunostained with anti‐IL‐1β and anti‐COX‐2 antibodies. (g) Quantification of data from e and f (*n* = 5 mice/group). Scale bar =100 μm (cortex, CA1, DG) and 200 μm (hippocampus). Data are presented as the mean ± *SEM* (*p < 0.05 and **p < 0.01 vs. vehicle)

Chronic neuroinflammation and microglial activation can prime and accelerate AD pathology and vice versa (Li et al., 2018). We previously observed that injection of ibrutinib (10 mg/kg; i.p. daily for 3 days) modulates gliosis and proinflammatory cytokine levels induced by LPS in the brains of wild‐type mice and in a microglial cell line (Nam et al., 2018). To assess whether ibrutinib modulates Aβ‐mediated gliosis and neuroinflammatory responses, 3‐month‐old 5xFAD mice were administered vehicle (5% DMSO +30% PEG +5% Tween‐80; i.p.) or ibrutinib (10 mg/kg; i.p.) by injection for 14 consecutive days, and brain sections were immunostained with anti‐Iba‐1, anti‐GFAP, and anti‐6E10 antibodies. In mice that received ibrutinib, Iba‐1 immunoreactivity in the cortex and hippocampus was significantly reduced (Figure 2a,c), and the number of Aβ plaques that colocalized with Iba‐1‐positive cells was significantly decreased in the hippocampal CA1 region (Figure 2a,d). GFAP immunoreactivity was significantly decreased in the hippocampus CA1 and DG but not cortex (Figure 2b,c), and the number of Aβ plaques that colocalized with GFAP‐positive cells was significantly reduced in the cortex and hippocampus (Figure 2b,d). A higher dose of ibrutinib (30 mg/kg; i.p. daily for 14 days) significantly reduced Iba‐1 and GFAP immunoreactivity in both cortex and hippocampus of 3‐month‐old 5xFAD mice (Figure S2a,b,e). However, in 6‐month‐old 5xFAD mice, no effects of 10 mg/kg ibrutinib (i.p. daily for 14 days) on Iba‐1 and GFAP immunoreactivity were observed (Figure S2c,d,f).

Next, we assessed Aβ‐induced proinflammatory cytokine levels. Three‐month‐old 5xFAD mice were administered vehicle (5% DMSO +30% PEG +5% Tween‐80; i.p.) or ibrutinib (10 mg/kg, i.p.) by injection for 14 consecutive days. Immunostaining of brain sections with anti‐IL‐1β and anti‐COX‐2 antibodies revealed that cortical and hippocampal IL‐1β and COX‐2 levels were significantly decreased in ibrutinib‐treated 3‐month‐old 5xFAD mice (Figure 2e–g). However, in 6‐ and 12‐month‐old 5xFAD mice, the same ibrutinib regimen did not alter proinflammatory cytokine levels in the brain (Figure S3a–e). Thus, the ability of ibrutinib to suppress glial activation and proinflammatory cytokine levels is restricted to 3‐month‐old 5xFAD mice.

### 2.3 Ibrutinib downregulates tau phosphorylation and tau kinase p‐CDK5 levels in 5xFAD mice

Hyperphosphorylation of tau is another histopathological hallmark of AD, and several recent studies have demonstrated that Aβ accumulation leads to tau phosphorylation and *vice versa* (Alonso et al., 2018; Wu et al., 2018). To assess tau phosphorylation levels, primary cortical neurons were incubated with ibrutinib (5 μM) or vehicle (1% DMSO) for 3 hr and immunoblotted with antibodies against AT8^Ser202 and Thr205^, AT180^Thr231^, Tau5, and β‐actin. Tau phosphorylation at Ser202/Thr205 was significantly decreased in primary cortical neurons treated with ibrutinib (Figure S4a–c). Next, to determine whether ibrutinib alters tau phosphorylation in a BTK‐dependent manner, primary cortical neurons were incubated with the BTK‐specific inhibitor CC‐292 (5 μM) or vehicle for 3 hr and immunoblotted. Interestingly, CC‐292 did not alter tau phosphorylation and total tau levels in primary cortical neurons (Figure S4d,e), suggesting that ibrutinib downregulates tau phosphorylation in a BTK‐independent manner.

**FIGURE 3:**
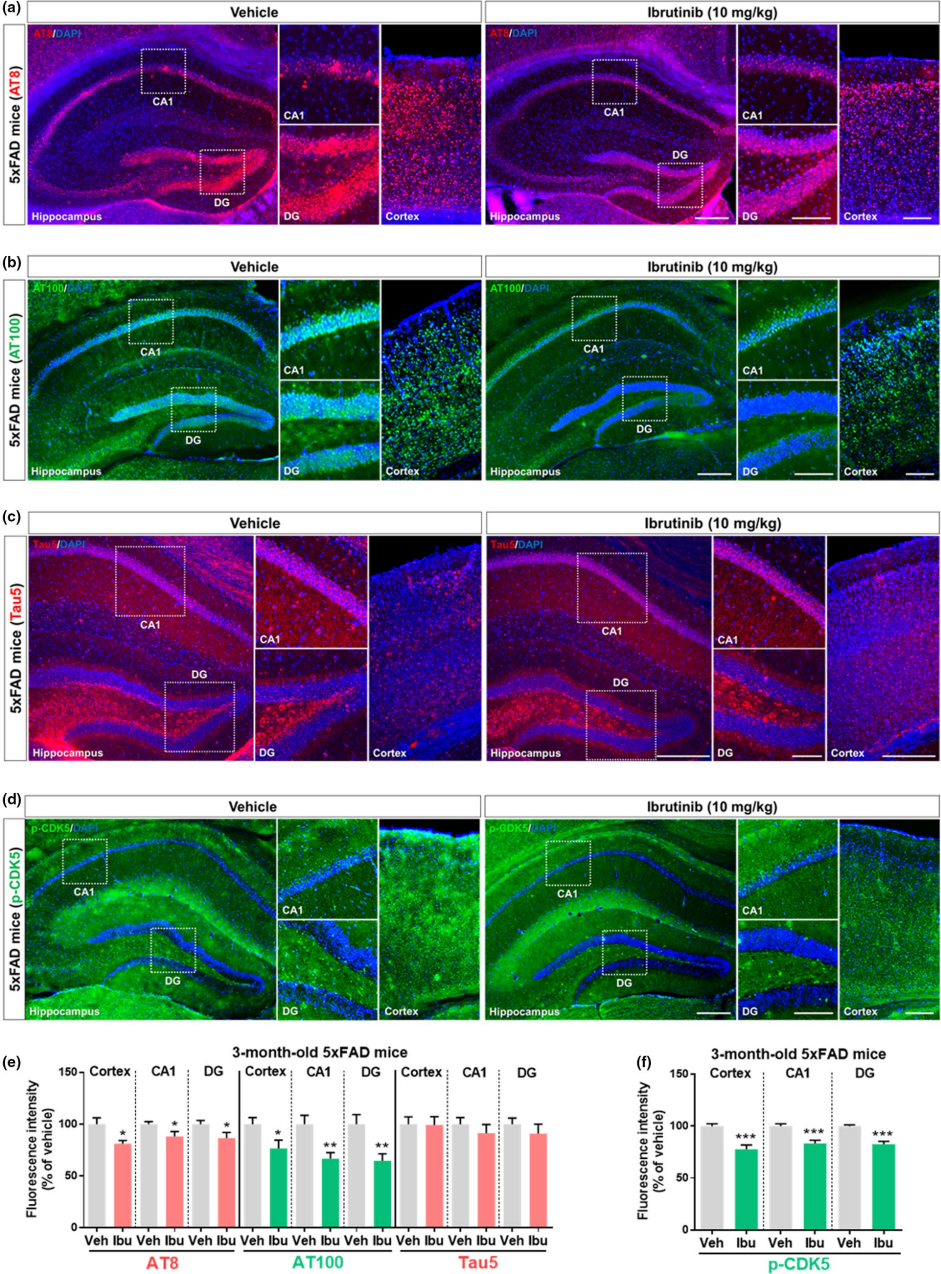
Ibrutinib significantly reduces tau phosphorylation and tau kinase p‐CDK5 levels in 3‐month‐old 5xFAD mice. (a and b) Ibrutinib or vehicle was injected (i.p.) daily for 14 consecutive days, and brain sections were immunostained with anti‐AT8 and anti‐AT100 antibodies. (c and d) Ibrutinib or vehicle was injected (i.p.) daily for 14 consecutive days, and brain sections were immunostained with anti‐tau5 and anti‐p‐CDK5 antibodies. (d) Quantification of data from a and b (*n* = 4–5 mice/group). (f) Quantification of data from c and d (*n* = 4–5 mice/group). Scale bar = 100 μm (cortex, CA1, DG) and 200 μm (hippocampus). Data are presented as the mean ± *SEM* (*p < 0.05, **p < 0.01, and ***p < 0.001 vs. vehicle)

Although NFTs are not observed in 5xFAD mice, we and others have reported that tau phosphorylation is detectable at appreciable levels in 3‐month‐old 5xFAD mice (Sawmiller et al., 2017). To assess tau phosphorylation, 3‐month‐old 5xFAD mice were administered vehicle or ibrutinib by injection (10 mg/kg; i.p. daily for 14 days). Ibrutinib significantly decreased tau phosphorylation at Ser202/Thr205 and Ser214/Thr212 (Figure 3a,b,e), but total tau levels were not altered compared with vehicle treatment (Figure 3c,f). A higher dose of ibrutinib (30 mg/kg; i.p. daily for 14 days) also significantly reduced tau phosphorylation at Ser214/Thr212 in the cortex and hippocampus in 3‐month‐old 5xFAD mice (Figure S5a,e). In 6‐month‐old 5xFAD mice, 10 mg/kg ibrutinib (i.p. daily for 14 days) again significantly decreased tau phosphorylation at Ser202/Thr205 and Ser214/Thr212 in the cortex and hippocampus (Figure S5b–c,f) without altering total tau levels (Figure S5d,f).

To further probe the mechanism by which ibrutinib modulates tau phosphorylation, we assessed tau kinase levels in 3‐month‐old 5xFAD mice via immunofluorescent staining with anti‐p‐CDK5 ^Tyr15^ and anti‐DYRK1A antibodies. Interestingly, ibrutinib (10 mg/kg; i.p. daily for 14 days) significantly downregulated CDK5 phosphorylation in the cortex and hippocampus (Figure 3d,f). Significant reductions of p‐CDK5 levels were also observed in 3‐month‐old 5xFAD mice injected with 30 mg/kg ibrutinib and 6‐month‐old 5xFAD mice injected with 10 mg/kg ibrutinib (Figure S6a,b,d (i.p. daily for 14 days)). By contrast, no effect of ibrutinib (10 mg/kg; i.p. daily for 14 days) on DYRK1A levels was observed in 3‐month‐old 5xFAD mice (Figure S6c,e). Thus, ibrutinib decreases tau phosphorylation in 5xFAD mice by reducing p‐CDK5 levels.

### 2.4 Ibrutinib suppresses micro‐ and astrogliosis and proinflammatory cytokine levels in PS19 mice

**FIGURE 4:**
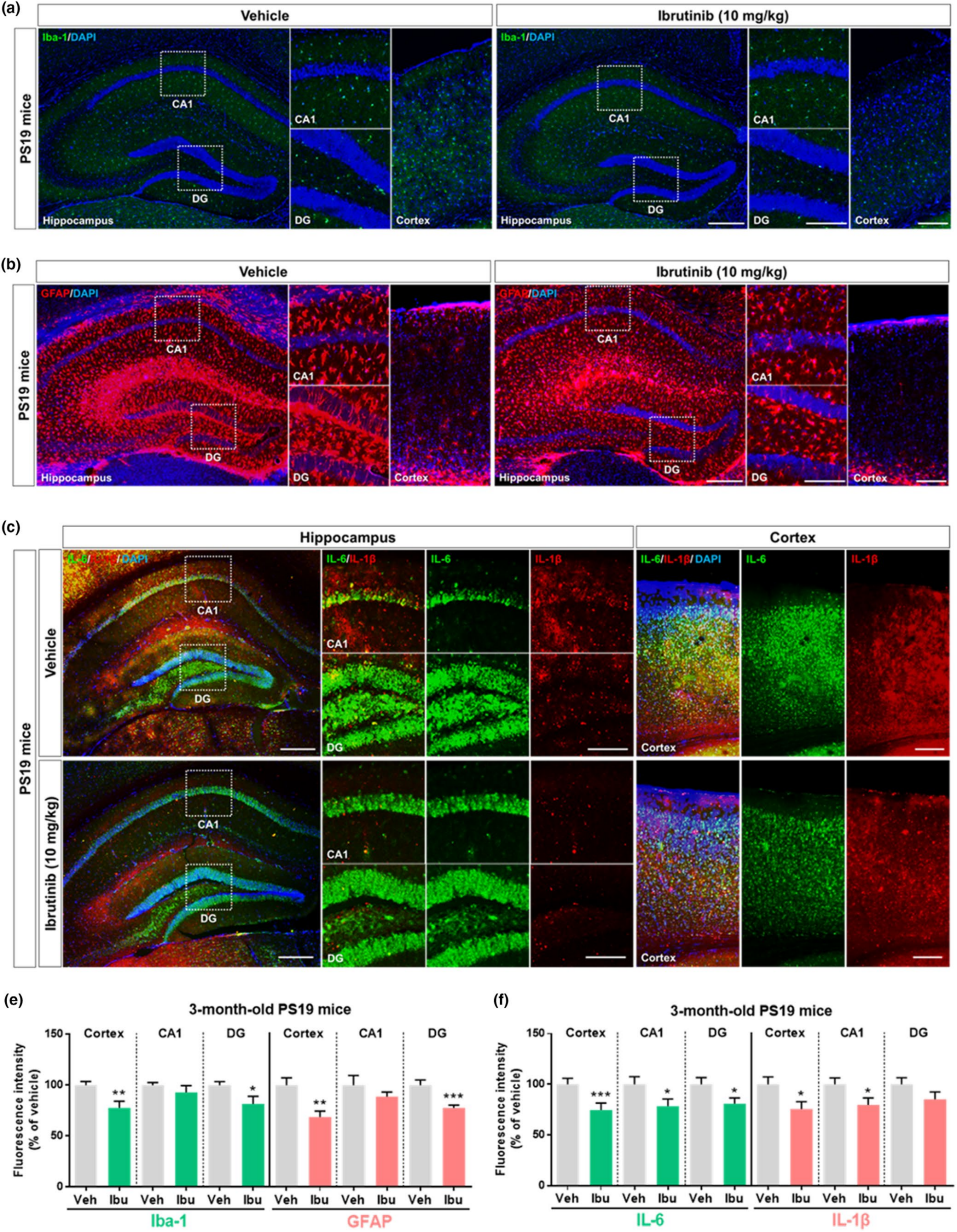
Ibrutinib alters tau‐induced glial activation and levels of the proinflammatory cytokines IL‐6 and IL‐1β in 3‐month‐old PS19 mice. (a and b) Ibrutinib or vehicle was injected (i.p.) daily for 14 consecutive days, and brain sections were immunostained with anti‐Iba‐1 and anti‐GFAP antibodies. (c) Ibrutinib or vehicle was injected (i.p.) daily for 14 consecutive days, and brain sections were immunostained with anti‐IL‐6 and anti‐IL‐1β antibodies. (e and f) Quantification of data from a to c (e, *n* = 3–5 mice/group; f, *n* = 4 mice/group). Scale bar = 100 μm (cortex, CA1, DG) and 200 μm (hippocampus). Data are presented as the mean ± *SEM* (*p < 0.05, **p < 0.01, and ***p < 0.001 vs. vehicle)

To further investigate tau‐mediated neuroinflammation, 3‐month‐old PS19 mice, a model of tauopathy, were administered vehicle (5% DMSO +30% PEG +5% Tween‐80; i.p.) or ibrutinib (10 mg/kg; i.p.) by injection daily for 14 consecutive days. Immunostaining of brain sections revealed that ibrutinib significantly downregulated Iba‐1 and GFAP immunoreactivity in the cortex and hippocampus DG region (Figure 4a,b,e). Furthermore, the immunoreactivities of the proinflammatory cytokines IL‐6, IL‐1β, and COX‐2 in the cortex and hippocampus were significantly lower in the mice that received ibrutinib (Figures 4c,f and S7a,b). These observations indicate that ibrutinib suppresses tau‐evoked gliosis and proinflammatory cytokine levels in 3‐month‐old PS19 mice.

### 2.5 Ibrutinib downregulates tau phosphorylation and tau kinase p‐CDK5 levels in PS19 mice

**FIGURE 5:**
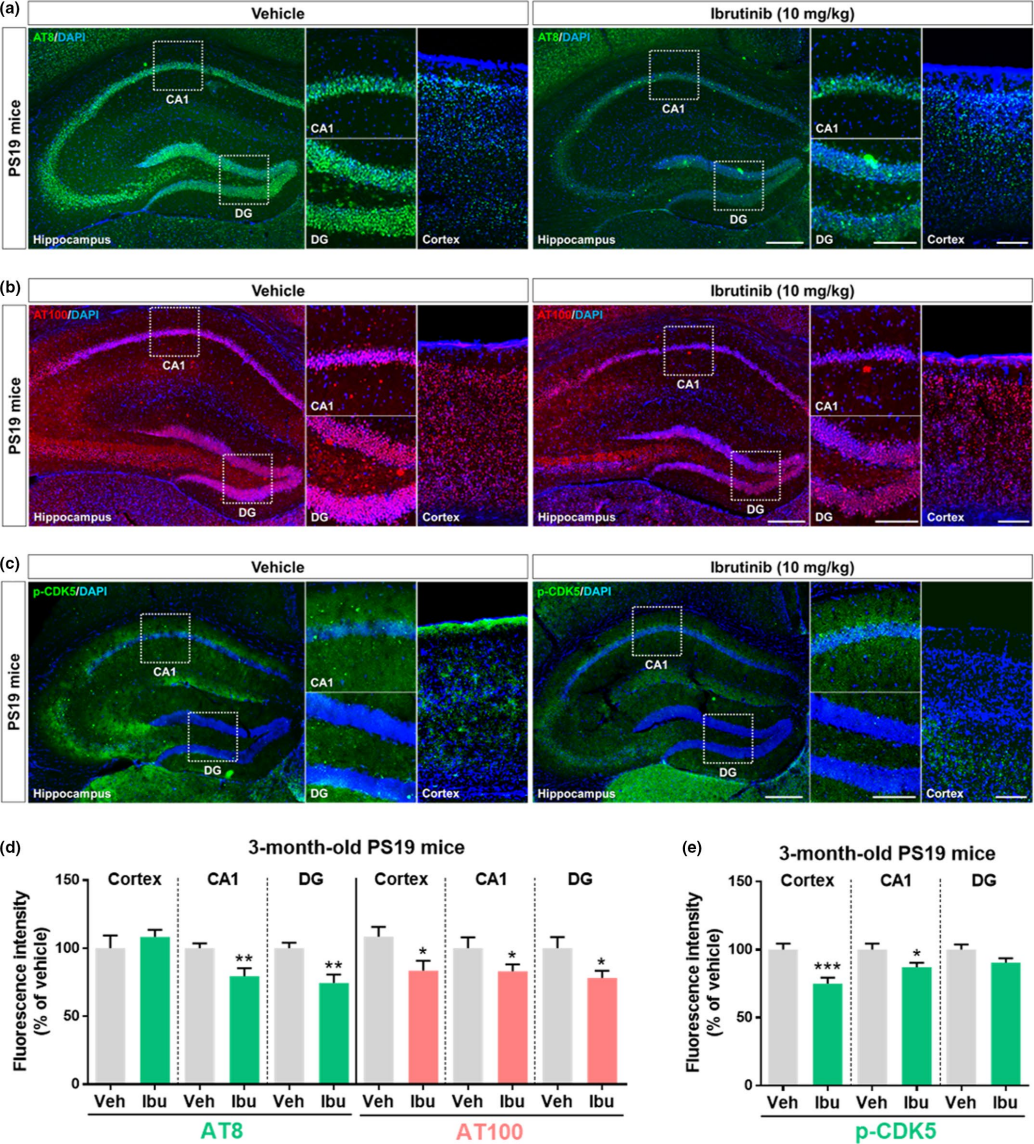
Ibrutinib reduces tau phosphorylation and tau kinase p‐CDK5 level in 3‐month‐old PS19 mice. (a and b) Ibrutinib or vehicle was injected (i.p.) daily for 14 consecutive days, and brain sections were immunostained with anti‐AT8 and anti‐AT100 antibodies. (c) Ibrutinib or vehicle was injected (i.p.) daily for 14 consecutive days, and brain sections were immunostained with an anti‐p‐CDK5 antibody. (d) Quantification of data from a and b (*n* = 3–5 mice/group). (e) Quantification of data from c (*n* = 4 mice/group). Scale bar = 100 μm (cortex, CA1, DG) and 200 μm (hippocampus). Data are presented as the mean ± *SEM* (*p < 0.05, **p < 0.01, and ***p < 0.001 vs. vehicle)

Given the effects of ibrutinib on tau‐induced neuroinflammation in PS19 mice, we next assessed tau phosphorylation and associated downstream signaling in this model (Jankowsky & Zheng, 2017). Immunostaining with antibodies against AT8^Ser202/Thr205^, AT100^Ser214/Thr212^, and Tau5 demonstrated that ibrutinib (10 mg/kg; i.p., daily for 14 days) significantly decreased tau phosphorylation at Ser202/Thr205 and Ser214/Thr212 in the hippocampus and/or cortex (Figure 5a,b,d) but not total tau levels (Figure S8a,b) compared with vehicle (5% DMSO +30% PEG +5% Tween‐80; i.p.). Furthermore, immunofluorescent staining with an anti‐p‐CDK5 ^Tyr15^ antibody to probe tau‐associated kinase activity showed that ibrutinib significantly downregulated p‐CDK5 in the cortex and hippocampus CA1 region (Figure 5c,e). Thus, consistent with the observations in 5xFAD mice, ibrutinib modulates tau phosphorylation in PS19 mice by reducing p‐CDK5 levels.

### 2.6 Ibrutinib improves long‐term memory and spinogenesis in 5xFAD mice

**FIGURE 6:**
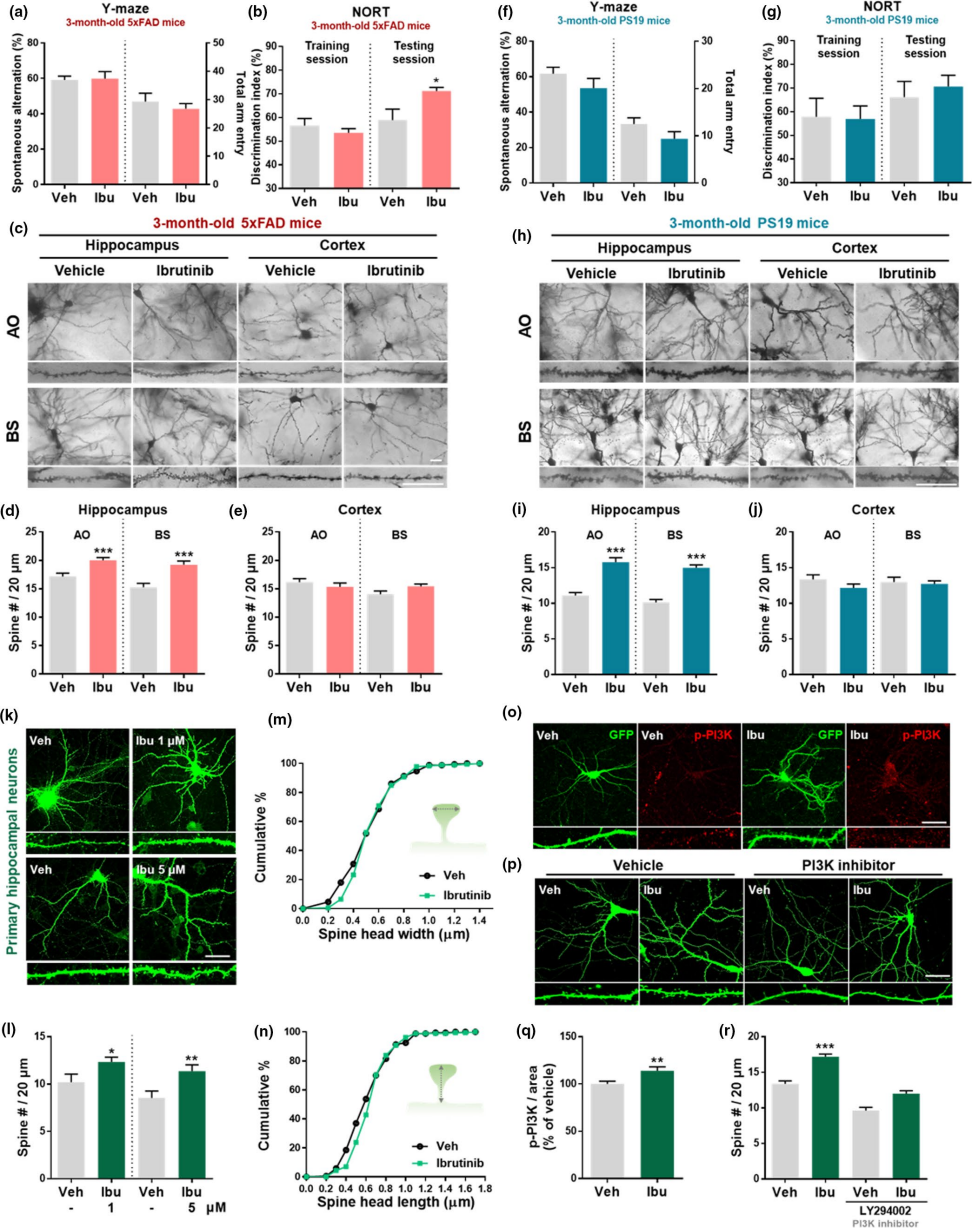
Ibrutinib improves long‐term memory and spinogenesis in vivo and in vitro. (a–b and f–g) Three‐month‐old 5xFAD and PS19 mice were injected (i.p.) with ibrutinib or vehicle daily for 14 consecutive days, and behavior tests were performed (*n* = 8–10 mice/group). (c and h) Three‐month‐old 5xFAD and PS19 mice were injected (i.p.) with ibrutinib or vehicle daily for 14 consecutive days, and brain sections were stained using the Golgi staining method. (d–e and i–j) Quantification of data from c and h (*n* = 4–5 mice/group). (k) Primary hippocampal neurons transfected with plasmid DNA encoding GFP were exposed to ibrutinib (1 or 5 μM) or vehicle (1% DMSO) for 24 hr, and dendritic spine number, width, and length were measured. (l–n) Quantification of data from k (n = 15–54 dendrites derived from individual neurons). (o) Primary hippocampal neurons transfected with plasmid DNA encoding GFP were treated with ibrutinib (5 μM) or vehicle for 24 hr and immunostaining with anti‐p‐PI3K antibody. (p) Primary hippocampal neurons transfected with plasmid DNA encoding GFP were treated sequentially with LY294002 (5 μM) for 1 hr and ibrutinib (5 μM) or vehicle (1% DMSO) for 23 hr. Immunostianing with an anti‐GFP antibody and dendritic spine number measurements were then performed. (q and r) Quantification of data from o and p (q, r = 29–34 dendrites derived from individual neurons). Scale bar = 20 μm (c,h) and 50 μm (k,o,p). Data are presented as the mean ± *SEM* (*p < 0.05, **p < 0.01, and ***p < 0.001 vs. vehicle)

Given ibrutinib's effects on Aβ‐ and tau‐induced neuroinflammation, amyloid plaques, and tau phosphorylation (Figures 1, 2, 3, 4, 5), we evaluated the behavioral performance of 3‐month‐old 5xFAD and PS19 mice injected with ibrutinib (10 mg/kg; i.p.) or vehicle daily for 14 days by conducting Y‐maze and novel object recognition (NOR) tests (Webster et al., 2014). Administration of ibrutinib did not affect spontaneous alterations in the Y‐maze test by either 5xFAD or PS19 mouse models (Figure 6a,f). Interestingly, ibrutinib significantly increased the preference for the novel object among 3‐month‐old 5xFAD mice but not 3‐month‐old PS19 mice (Figure 6b,g). Oral administration of 30 mg/kg ibrutinib (p.o. daily for 30 days) also significantly increased the preference of 3‐month‐old 5xFAD mice for the novel object but did not alter spontaneous alterations in the Y‐maze test (Figure S9a,b). In 6‐month‐old 5xFAD mice, ibrutinib injection (10 mg/kg; i.p.) had no effect on spontaneous alterations and novelty preference (Figure S9c,d). These data suggest that ibrutinib affects long‐term memory formation in 3‐month‐old 5xFAD mice (early stage of Aβ pathogenesis).

To elucidate the mechanism by which ibrutinib improves long‐term memory, dendritic spine number was analyzed. Golgi staining revealed that ibrutinib injection significantly increased apical oblique (AO) and basal shaft (BS) dendritic spine numbers in the hippocampus in 3‐month‐old 5xFAD and PS19 mice (Figure 6c–e,h–j). Oral administration of ibrutinib (30 mg/kg; p.o. daily for 30 days) also upregulated AO and BS dendritic spine numbers in the hippocampus in 3‐month‐old 5xFAD mice (Figure S9e–g).

Ibrutinib's effects on dendritic spine number *in vitro* were investigated in primary hippocampal neurons transfected with plasmid DNA encoding GFP. Treatment with ibrutinib (1 or 5 μM) for 24 hr significantly increased the number of dendritic spines in primary hippocampal neurons but did not alter spine head width and length compared with treatment with vehicle (1% DMSO) (Figure 6k–n). Moreover, the BTK‐specific inhibitor CC‐292 (1 or 5 μM) did not alter dendritic spinogenesis in primary hippocampal neurons (Figure S10a,b), suggesting that ibrutinib upregulates dendritic spine number in a BTK‐independent manner.

We then evaluated the influence of ibrutinib on PI3K phosphorylation, which is involved in dendritic spinogenesis and synaptic function (Sanchez‐Alegria et al., 2018). Immunostaining of GFP‐transfected primary hippocampal neurons with an anti‐p‐PI3K antibody revealed that ibrutinib treatment (5 μM) significantly upregulated PI3K phosphorylation compared with vehicle (1% DMSO) (Figure 6o, q). To examine the role of PI3K signaling pathways in ibrutinib's effects on dendritic spinogenesis, primary hippocampal neurons were first treated with the PI3K inhibitor LY294002 (5 μM) or vehicle for 1 hr and then treated with ibrutinib (5 μM) or vehicle (1% DMSO) for 23 hr. Ibrutinib was unable to enhance the dendritic spine number in primary hippocampal neurons pretreated with LY294002 (Figure 6p,r). Consistent with the effects observed in vitro, ibrutinib (10 mg/kg; i.p. daily for 14 days) significantly enhanced p‐PI3K levels in 3‐month‐old PS19 mice (Figure S11a,b). These data suggest that ibrutinib upregulates synaptic function via PI3K phosphorylation in PS19 mice.

## 3 DISCUSSION

Ibrutinib irreversibly inhibits the auto‐phosphorylation of Tyr^223^ of BTK and the auto‐phosphorylation of other kinases, including EGFR, BMX, and JAK3 (Davids & Brown, 2014). Pharmacokinetic analyses of human plasma and cerebrospinal fluid and distribution analyses of mouse brain tissue have demonstrated that ibrutinib penetrates the BBB (Bernard et al., 2015; Mason et al., 2017). Here, we confirmed that ibrutinib crosses the BBB in WT mice (Table S1). Following on our recent finding that ibrutinib significantly reduces LPS‐induced neuroinflammation in vitro and in vivo (Nam et al., 2018), in the present study, we evaluated whether ibrutinib affects AD pathology and synaptic/cognitive function in animal models of AD (5xFAD and PS19 mice) and primary hippocampal neurons. In addition to significantly reducing cortical and hippocampal Aβ accumulation in 5xFAD mice (Figure 1), ibrutinib injection downregulated proinflammatory cytokines and alleviated neuroinflammation in 5xFAD and PS19 mice (Figures 2 and 4). In both mouse models, ibrutinib reduced the phosphorylation of tau and levels of the tau‐related kinase p‐CDK5 (Figures 3 and 5). Moreover, ibrutinib improved long‐term memory and the number of hippocampal synapses in 5xFAD mice, and studies in primary hippocampal neurons demonstrated that the induction of dendritic spinogenesis by ibrutinib was PI3K‐dependent (Figure 6).

Aβ, which is derived from APP, is a small protein of 36–43 amino acids and is the major pathogenic component in AD (O'Brien & Wong, 2011). Our study is the first to document that intraperitoneal and oral injection of ibrutinib significantly reduces Aβ accumulation in 3‐ and 6‐month‐old 5xFAD mice. Specifically, intraperitoneal injection of ibrutinib suppressed Aβ deposition in both hippocampus CA1 and DG in 3‐month‐old 5xFAD mice but only hippocampus CA1 in 6‐month‐old 5xFAD mice. It has been reported that Aβ burden is higher in the CA1 region than the DG region in 6‐ and 12‐month‐old AD mice (Reilly et al., 2003). These differences in Aβ load may underlie the observed brain region‐ and age‐specific effects of ibrutinib on Aβ plaque regulation in 3‐ and 6‐month‐old 5xFAD mice. However, a regimen of daily injections for 2 weeks is not sufficient to modulate Aβ pathology, and thus, further studies using longer treatment durations or higher doses of ibrutinib are needed to determine whether ibrutinib differentially modulates brain region‐ and/or age‐dependent Aβ deposition in 6‐month‐old 5xFAD mice. In addition, intraperitoneal administration of ibrutinib decreased Aβ deposition in the both hippocampus CA1 and DG, whereas oral administration affects Aβ deposition only in hippocampus DG of 3‐month‐old 5xFAD mice. This result indicated that the way of injection route might differentially influence on the brain distribution of drug, thereby exhibiting brain region‐specific effect on the regulation of Aβ pathogenesis. There was no effect of ibrutinib in 12‐month‐old 5xFAD mice, which feature high accumulation of Aβ (Figures 1a–c and S1). In H4 cells overexpressing APP, ibrutinib increased sAPPα levels and decreased FL‐APP and APP‐CTF levels (Figure 1d,e). These observations indicate that ibrutinib reduces Aβ accumulation by increasing α‐secretase activity and decreasing γ‐secretase activity in APP processing. It is possible that ibrutinib affects Aβ plaque burden via multi‐directional pathways. For instance, ibrutinib may directly and/or indirectly interact with EGFR to inhibit EGFR signaling and therefore modulate Aβ pathology. Consistent with this notion, we recently reported that the EGFR inhibitor regorafenib significantly reduces Aβ pathogenesis, suggesting that EGFR might be a potential off‐target of ibrutinib for Aβ inhibition (Han et al., 2020). Another possibility is that ibrutinib regulates Aβ‐degrading enzymes such as neprilysin and insulin‐degrading enzyme to reduce Aβ accumulation. Future studies will examine whether ibrutinib regulates Aβ pathogenesis via BTK and/or other off‐target molecules.

Alzheimer's disease and neuroinflammation are closely related and form a vicious cycle in which one increases the pathological burden of the other (Akiyama et al., 2000; Cai et al., 2014). In line with our previous report (Nam et al., 2018), administration of ibrutinib was associated with significantly lower microgliosis (Iba‐1) and astrogliosis (GFAP) in the brains of 5xFAD mice (Figures 2a–c and S2a,b,e). Ibrutinib also reduced the numbers of Aβ plaques that colocalized with microglia and astrocytes (6E10), as well as proinflammatory cytokines in 5xFAD mice (Figure 2e–g). However, the anti‐inflammatory effects of ibrutinib vanished in 5xFAD mice older than 6 months of age (Figures S2c,d,f and S3). How does ibrutinib regulate Aβ and tau‐mediated neuroinflammatory responses in mouse models of AD? Interestingly, we and others have reported that the EGFR inhibitors regorafenib and dasatinib downregulate peripheral and central inflammation in wild‐type mice and 5xFAD mice (Han et al., 2020; Liu et al., 2020; Ryu et al., 2019). These reports suggest that ibrutinib might suppress Aβ‐ and tau‐evoked neuroinflammation in mouse models of AD via EGFR and BTK inhibition. Of course, it is possible that other off‐targets of ibrutinib (including JAK3 and BMK) are involved in these processes; in future work, we will determine which ibrutinib target(s) and/or off‐target(s) underlie the effects of ibrutinib on Aβ‐ and/or tau‐induced peripheral and central inflammation in 5xFAD and PS19 mice. Overall, our results suggest that ibrutinib alleviates the early stage of Aβ‐ and tau‐induced neuroinflammation by downregulating inflammatory cytokines.

Abnormal hyperphosphorylation of tau is another histopathological hallmark of AD (Alonso et al., 2018). Remarkably, administration of ibrutinib in 3‐ and 6‐month‐old 5xFAD mice significantly reduced tau phosphorylation at AT8 (Ser^202^/Thr^205^) and AT100 (Thr^212^/Ser^214^) and downregulated p‐CDK5 levels (Figures 3a,b,d–f, S5a–c,e,f and S6a,b,d). In addition, administration of ibrutinib in 3‐month‐old PS19 mice significantly reduced tau phosphorylation at AT8 (Ser^202^/Thr^205^) and AT100 (Thr^212^/Ser^214^). Interestingly, we observed that AT100 levels were significantly reduced in the hippocampus and cortex of PS19 mice, whereas AT8 levels were only decreased in the hippocampus of PS19 mice (Figure 5). How does ibrutinib differentially regulate tau phosphorylation according to brain region in PS19 mice? We assume that daily injections for 2 weeks and/or intraperitoneal injection of 10 mg/kg were not sufficient to affect tau phosphorylation in the cortex, and thus, studies verifying brain region‐specific tau phosphorylation using longer regimens (i.e., daily 4 weeks, 8 weeks) and/or higher doses of ibrutinib (i.e., 30 mg/kg) are needed. Another possibility is due to brain region‐specific tauopathy. A recent study demonstrated that tauopathy differs in hippocampal subregions (i.e., CA1, DG, CA3), indicating that ibrutinib might differentially regulate tau phosphorylation at Ser^202^/Thr^205^ in brain region‐specific manner in PS19 mice (Boluda et al., 2015). Surprisingly, we found that ibrutinib affects tau phosphorylation in a BTK‐independent manner (Figure S4). Importantly, we recently demonstrated that the EGFR inhibitor regorafenib significantly reduces tau phosphorylation at Thr^212^/Ser^214^ (Han et al., 2020). Based on the literature and our findings, we hypothesize that ibrutinib decreases tau pathology through EGFR inhibition, but other off‐targets of ibrutinib may be associated with these processes. Therefore, the molecular mechanism by which ibrutinib's off‐targets (e.g., EGFR, JAK3, BMX) modulate tauopathy will be explored in a future study.

CDK5 is not only closely correlated with AT8 in NFTs (late stage of tau pathology) but also contributes to tau phosphorylation at AT8 (Ser^202^/Thr^205^) and AT100 (Thr^212^/Ser^214^) in the early stage of tau pathology (Augustinack et al., 2002; Castro‐Alvarez et al., 2014). In addition, CDK5 induces hyperphosphorylation of tau and enhances tau aggregation in p25 (CDK5 activator) and h‐P301L double‐Tg mice (Noble et al., 2003), and exposure of 3xTg mice (a model of AD with both Aβ and tau pathology) to LPS increases tau phosphorylation via CDK5 activation (Kitazawa et al., 2005). Several studies have demonstrated that the tau kinase CDK5 can contribute to Aβ pathology and tau phosphorylation (Cruz et al., 2006). For instance, treatment of rat hippocampal cells with fibrillary Aβ significantly increases the activity and level of CDK5, and a CDK5 inhibitor protects brain cells from Aβ‐induced cell death (Alvarez et al., 1999). Interestingly, Aβ levels in cultured neurons are remarkably reduced by inhibition of APP phosphorylation at Thr^668^ using CDK5 inhibitors or expression of the APP^T668A^ mutant (M.‐S. Lee et al., 2003). These findings suggest that the reduction of p‐CDK5 levels by ibrutinib may affect not only tau phosphorylation but also Aβ‐related pathology. Consistent with the significant decreases in tau phosphorylation in 5xFAD mice (Aβ‐overexpressing mice), ibrutinib administration significantly reduced phosphorylation of tau (AT8/AT100) and CDK5 and neuroinflammation (Iba‐1/GFAP) and inflammatory cytokine levels (IL‐6/IL‐1β/COX2) in PS19 mice (tau‐overexpressing mice) (Figures 4, 5 and S7). Of course, ibrutinib may affect other tau kinases to alter tau pathology (e.g., p‐GSK3β, PKA, PKC, CAMKII). Further study will reveal whether ibrutinib age‐ and/or dose‐dependently modulates tauopathy in AD mouse models and the specific underlying mechanisms.

Neuroinflammation can lead to synaptic dysfunction and memory disorders, which eventually contribute to neurodegeneration (Guzman‐Martinez et al., 2019). Given the effects of ibrutinib on LPS‐, Aβ‐, and tau‐mediated neuroinflammatory responses, we assumed that ibrutinib affects learning and memory, which we assessed by conducting behavior experiments using the Y‐maze and NOR tests. Although both the Y‐maze and NOR tests analyze hippocampus‐dependent cognitive function, the Y‐maze test evaluates short‐term spatial learning memory (Kraeuter et al., 2019), whereas the NOR test focuses on long‐term recognition memory (Antunes & Biala, 2012). Considering the pivotal role of the hippocampus in consolidating short‐term memories into long‐term context‐dependent episodic memories (Lisman & Grace, 2005), the NOR test is a more sensitive behavioral paradigm for evaluating hippocampus‐dependent long‐term memory. In the present study, we found that ibrutinib improved long‐term memory (but not short‐term memory) in 3‐month‐old 5xFAD mice. Surprisingly, ibrutinib did not improve short‐ or long‐term memory in 6‐month‐old 5xFAD mice and 3‐month‐old PS19 mice (Figures 6 and S9), suggesting that ibrutinib improves hippocampal long‐term recognition memory but not short‐term spatial memory in the early stage of Aβ pathogenesis. Consistent with the lack of effects of ibrutinib on neuroinflammation in 6‐month‐old 5xFAD mice, administration of ibrutinib did not improve cognitive function in these mice with moderate Aβ pathogenesis (Figure S9). Taken together, these findings indicate that the effects of ibrutinib on cognitive function are restricted to the early stage of Aβ pathology and are lost once Aβ pathogenesis reaches moderate levels.

Correlations between dendritic spine number and memory behavior have been observed in various disease models, including models of AD (Roy et al., 2016). Interestingly, administration of ibrutinib increased the number of dendritic spines in the hippocampus in both 5xFAD and PS19 mice (Figure 6). To verify the effect of ibrutinib on hippocampal spinogenesis, we analyzed the number of spines per length and spine morphology in primary hippocampal neurons treated with ibrutinib or BTK‐specific inhibitor CC292. Importantly, the number of spines per length was enhanced by ibrutinib but not CC‐292 (Figure 6), indicating that ibrutinib modulates spinogenesis through a BTK‐independent pathway. Consistent with these results, hippocampus‐specific enhanced spinogenesis was observed in ibrutinib‐administered 5xFAD mice and PS19 mice (Figure 6). We showed that ibrutinib promoted dendritic spine number through the PI3K signaling pathway, which is an important signaling pathway for cognitive and synaptic function in mammalian brains (Sanchez‐Alegria et al., 2018). In addition, PI3K activity may regulate APP processing to non‐amyloidogenic pathways and induce the secretion of insulin‐degrading enzyme, which contributes to the degradation of Aβ aggregates (Shieh et al., 2020). PI3K may also affect tau pathology by inhibiting GSK3β‐induced tau phosphorylation through the PI3K‐AKT pathway (Kitagishi et al., 2014). These reports suggest that upregulation of PI3K phosphorylation by ibrutinib is not only essential for dendritic spinogenesis but may also contribute to Aβ/tau‐related pathology. Based on the literature and our findings, ibrutinib may modulate cognitive/synaptic function via other spinogenesis‐related signaling pathways (e.g., Ras, RAP signaling). In future work, we will further dissect the molecular mechanisms by which ibrutinib enhances dendritic spine formation in mouse models of AD.

In summary, we have demonstrated that ibrutinib alters Aβ accumulation, neuroinflammation, and tau phosphorylation in 5xFAD mice. Ibrutinib‐injected 5xFAD mice exhibit improved long‐term memory and increases in dendritic spine number. In addition, ibrutinib modulates tauopathy and tau‐mediated neuroinflammation and enhances spinogenesis in a PI3K‐dependent manner in PS19 mice. Taken together, our results suggest that ibrutinib holds therapeutic potential for modulating early‐stage AD‐related pathologies.

## 4 EXPERIMENTAL PROCEDURES

### 4.1 Animals

We used 5xFAD and PS19 mice as mouse models of AD (Oakley et al., 2006; Takeuchi et al., 2011). 5xFAD mice carry five familial AD mutations (APP^Sew,Lon,Flo^ and PS1^M146L,L286V^) under the Thy1 promoter, resulting in overexpression of Aβ. PS19 mice are models of tauopathy that carry the hP301S mutation, which comprises one N‐terminal insert and four microtubule binding repeats (1N4R), under the mouse prion protein promoter. 5xFAD (Stock No. 34848‐JAX; B6. Cg‐Tg (APPSwFlLon,PSEN1*M146L*L286V)6799Vas/Mmjax) and PS19 (Stock No. 008169; B6;C3‐Tg (Prnp‐MAPT*P301S)PS19Vle/J) mice were purchased from Jackson Laboratory (Bar Harbor, ME, USA). Genotyping of each strain was performed using genomic DNA extracted from a tail snip. Only male mice were used in the experiments to minimize the effect of hormones in the behavioral analysis. All animal experiments were performed in accordance with approved animal protocols and guidelines established by the Korea Brain Research Institute Animal Care and Use Committee (IACUC‐19‐00042, IACUC‐19‐00049).

### 4.2 Drug administration

Ibrutinib (S2680; Selleck Chemicals, Houston, TX, USA) was reconstituted in vehicle (5% DMSO +30% PEG300 + 5% Tween‐80) at the specified dosage. 5xFAD (3‐, 6‐, and 12‐month‐old) and PS19 (3‐month‐old) mice were intraperitoneally injected with 10 mg/kg or 30 mg/kg ibrutinib daily for 14 days. For oral administration, 30 mg/kg ibrutinib was administered to 3‐month‐old 5xFAD mice daily for 30 days.

### 4.3 Cell culture

APP‐H4 cells are a line of H4 cells that produce high levels of Aβ due to overexpression of hAPP. Cells were maintained in high‐glucose DMEM supplemented with 10% FBS and gentamycin in a 5% CO_2_ incubator.

### 4.4 APP processing in APP‐H4 cells

After treatment with vehicle (1% DMSO) or ibrutinib (5 μM) for 24 hr, APP‐H4 cells were homogenized in RIPA buffer (150 mM sodium chloride, 1% Triton X‐100, 0.5% sodium deoxycholate, 0.1% SDS, 50 mM Tris, pH 8.0) with cOmplete™ protease inhibitor cocktail (Roche, Basel, Switzerland) and PhosSTOP™ (Roche). The supernatant was harvested to measure sAPPα, and the cell lysate was harvested to measure FL‐APP and APP‐CTFs. The detergent‐compatible protein assay (Bio‐Rad, Hercules, CA, USA) was performed to quantify the protein concentrations, and equal amounts of protein were loaded onto 8% SDS‐PAGE gels.

### 4.5 Behavioral tests

The Y‐maze test was conducted to measure short‐term spatial memory. A mouse was placed in one of three arms (35 cm × 7 cm × 15 cm) of the maze, which met at an angle of 120°, and allowed to explore freely for 5 min. Spontaneous alternations were recorded and analyzed by using a video camera connected to tracking software (EthoVision XT, Noldus, Wageningen, the Netherlands). The percentage of spontaneous alternations was calculated by dividing the number of successful alternations (e.g., ABC and BCA but not CCA) by the total number of alternation triads.

The novel object recognition (NOR) test was conducted to analyze long‐term memory. Each mouse was allowed a 5‐min training phase with two identical objects in an open‐field box (50 cm × 50 cm × 30 cm). The apparatus and the objects were thoroughly swabbed with 70% ethanol between trials to eliminate odor cues. Twenty‐four hours later, the mice underwent a 5‐min retention testing phase in the same apparatus with one familiar object and one novel object. The location of the two objects was counterbalanced in the arena. Each trial was recorded, and the exploration time was manually counted. Behavior was considered exploratory when the mouse's nose was pointed toward the object. The relative exploration time was calculated to assess the object preference (%) as follows: [Discrimination index (%) of object = Novel^time^/(Familiar^time^ + Novel^time^) × 100].

### 4.6 Brain tissue preparation and immunohistochemistry

Paraformaldehyde (PFA) solution (4%) was used to perfuse and fix the mice 6 hr after the last administration of ibrutinib. A Leica CM1850 cryostat (Leica Biosystems, Buffalo Grove, IL, USA) was used to prepare brain sections with a thickness of 30 μm. The sections were incubated in staining solution (0.5 mg/ml bovine serum albumin and 0.3% Triton X‐100 in PBS) with the 1° antibodies at 4°C overnight. After washing 4 times with PBS, the sections were incubated with the 2° antibody at room temperature for 1 hr (see Table S2 for detailed information on the antibodies used). After washing 4 times with PBS, the sections were mounted with VECTASHIELD^®^ Antifade Mounting Medium with DAPI (Vector Laboratories), and images were acquired using a DMi8 inverted fluorescence microscope (Leica Microsystems, Wetzlar, Germany) and analyzed using the ImageJ software (version 1.53a, US National Institutes of Health, Bethesda, MD, USA).

### 4.7 Golgi staining

To assess the formation of dendritic spines in vivo, Golgi staining was conducted using an FD Rapid GolgiStain Kit (FD Neurotechnologies, Ellicott City, MD, USA) according to the manufacturer's procedure.

### 4.8 Primary neuronal culture and immunocytochemistry

To examine spinogenesis, primary hippocampal and cortical cells were cultured from Sprague Dawley rat embryos (day 18). The primary hippocampal/cortical neurons were transfected with plasmid DNA encoding green fluorescent protein (GFP) using Lipofectamine 2000 and treated with vehicle (1% DMSO) or ibrutinib (1 or 5 μM) for 24 hr. To investigate the role of PI3K in the modulation of dendritic spine formation by ibrutinib, GFP‐transfected primary hippocampal neurons were pretreated with 5 μM PI3K inhibitor (LY294002; Calbiochem, San Diego, CA, USA) or vehicle (1% DMSO) for 1 hr before treatment with 5 μM ibrutinib or vehicle (1% DMSO) for 23 hr. After fixation for either 8 min in methanol or 10 min in 4% paraformaldehyde and washing thrice with PBS, the treated neurons were incubated overnight with anti‐p‐PI3K (p85^Y458^/p55^Y199^) (1:200; Cell Signaling, Danvers, MA, USA) antibodies in GDB buffer (Nam et al., 2018) at 4°C. After washing thrice with PBS, the neurons were incubated for 1 hr with Alexa Fluor 488‐conjugated anti‐mouse and Alexa Fluor 555‐conjugated anti‐rabbit (1:400; Molecular Probes, Eugene, OR, USA) at room temperature. Images of cells mounted with VECTASHIELD^®^ Antifade Mounting Medium with DAPI (Vector Laboratories) were captured from a single plane using an A1 Confocal Laser Microscope (Nikon, Tokyo, Japan), and ImageJ software was used to analyze dendritic spine density. For each sample, 6 to 10 individual images were assessed by a blinded reviewer.

### 4.9 Drug distribution analysis

Analysis of the distribution of ibrutinib in brain tissue was commissioned to the Daegu Gyeongbuk Medical Innovation Foundation (DGMIF). Briefly, wild‐type mice were injected with ibrutinib (10 mg/kg, i.p.) or vehicle daily for 14 consecutive days. The brain was extracted, and the brain hemispheres were weighed and homogenized in 400 μl of PBS. The brain homogenates were analyzed by Agilent 1290 high‐performance liquid chromatography (Agilent, USA) with a Kinetex C18 column and Triple Quad 5500 mass spectrometry (Applied Biosystems, USA).

### 4.10 Statistical analysis

GraphPad Prism 7 software (GraphPad Software, San Diego, CA, USA) was used for all data analyses. Unpaired two‐tailed *t* tests were used for comparisons between two groups, and one‐way ANOVA was used for multiple comparisons. Tukey's multiple‐comparison test was used for post hoc analyses. Data are presented as the mean ± *SEM* (*p < 0.05, **p < 0.01, and ***p < 0.001).

## CONFLICT OF INTEREST

The authors declare no conflict of interest.

## AUTHOR CONTRIBUTIONS

H.S.H., M.S., Y.H.K., and K.S. conceived and participated in the design of the study. H.L., S.G.K., and J.K. mainly wrote the manuscript, performed experiments, and confirmed all data analyses in all figures and supporting information. R.J.K. and S.M.K. participated in preliminary manuscript preparation and experiments. K.M.H., H.H.P., K.K., Y.M.S., and H.Y.N. performed molecular/cellular and in vivo experiments. All authors read and approved the final manuscript.

## Supporting information

Supplementary Material

## Data Availability

All data generated and/or analyzed during this study are included in this article, and the data that support the results of this study are available from the corresponding author upon reasonable request.
