# Corrigendum to: Ibrutinib modulates Aβ/tau pathology, neuroinflammation, and cognitive function in mouse models of Alzheimer's disease

**DOI:** 10.1111/acel.13953

**Published:** 2023-08-03

Hyun‐ju Lee, Seong Gak Jeon, Jieun Kim, Ri Jin Kang, Seong‐Min Kim, Kyung‐Min Han, HyunHee Park, Ki‐taek Kim, You Me Sung, Hye Yeon Nam, Young Ho Koh, Minseok Song, Kyoungho Suk, Hyang‐Sook Hoe. *Aging Cell*, 20(3), e13332. https://doi.org/10.1111/acel.13332

In the published version of Lee et al. (2021), the authors noticed that the representative image of hippocampal BS in vehicle‐treated PS19 mice was inadvertently duplicated as the representative image of cortical BS in Figure 6h. We have corrected the representative image of cortical BS in vehicle‐treated PS19 mice in Figure 6h below (bottom line, third panel).

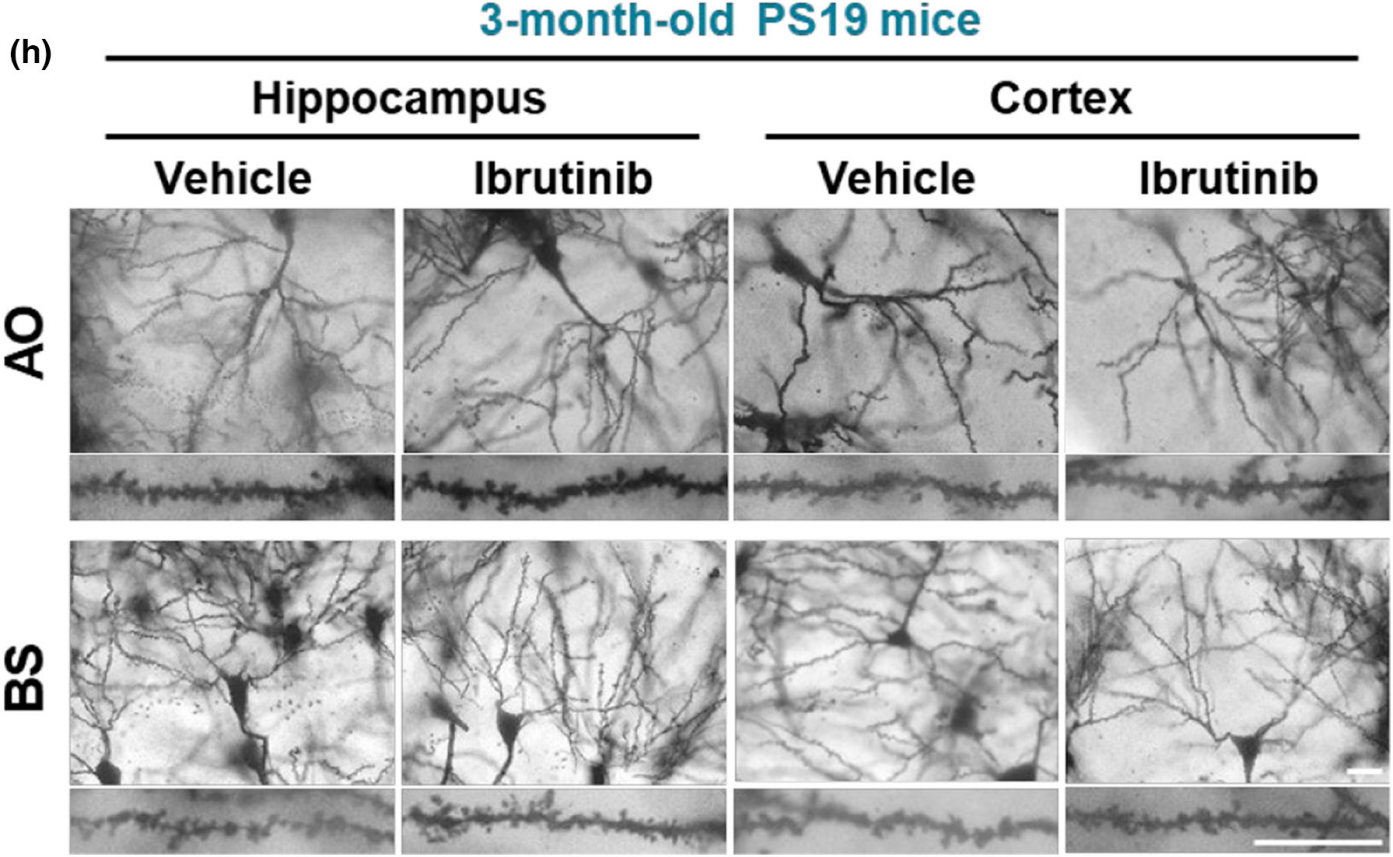

The authors would like to apologize for the inconvenience caused.
