# Supplementary material for: Ibrutinib modulates Aβ/tau pathology, neuroinflammation, and cognitive function in mouse models of Alzheimer's disease
Source: Aging Cell. 2021 Mar 11;20(3):e13332. doi: 10.1111/acel.13332 (PMC7963331; doi:10.1111/acel.13332)
Supplement: Supplementary file 1 — Supplementary Material [file ACEL-20-e13332-s001.docx]

**Supporting Information**

**Ibrutinib modulates Aβ/tau pathology, neuroinflammation, and cognitive function in mouse models of Alzheimer’s disease**

**Hyun-ju Lee^1,*^ | Seong Gak Jeon^1,*^ | Jieun Kim^1,*^ | Ri Jin Kang^1,*^ | Seong-Min Kim^1^ | Kyung-Min Han^1^ | HyunHee Park^1^ | Ki-taek Kim^2^ | You Me Sung^3^ | Hye Yeon Nam^1^ | Young Ho Koh^4^ | Minseok Song^2^ | Kyoungho Suk^5^ | Hyang-Sook Hoe^1,6,†^**

^1^Department of Neural Development and Disease, Korea Brain Research Institute (KBRI), 61, Cheomdan-ro, Daegu, Republic of Korea

^2^Department of Life Sciences, Yeungnam University, Gyeongsan, Gyeongsanbuk-do, 38541, Republic of Korea

^3^Korea Mouse Phenotyping Center (KMPC), Seoul National University, Seoul, Republic of Korea

^4^Center for Biomedical Sciences, Center for Infectious Diseases, Division of Brain Disease, Korea National Institute of Health, Heungdeok-gu, Republic of Korea

^5^Department of Pharmacology, Brain Science & Engineering Institute, School of Medicine, Kyungpook National University, Daegu, Republic of Korea

^6^Department of Brain and Cognitive Sciences, Daegu Gyeongbuk Institute of Science & Technology, Daegu 42988, Korea

**Supporting Information list**

**1 | Supplementary figures**

**FIGURE S1 Ibrutinib suppresses Aβ plaque levels in 3- and 6-month-old 5xFAD mice**

**FIGURE S2 Ibrutinib downregulates microglial and astrocyte activation in 3-month-old but not 6-month-old 5xFAD mice**

**FIGURE S3 Proinflammatory cytokine levels in 6- and 12-month-old 5xFAD mice are not altered by ibrutinib**

**FIGURE S4 Tau phosphorylation is suppressed by ibrutinib but not by the BTK-specific inhibitor CC-292**

**FIGURE S5 Ibrutinib reduces tau phosphorylation in 3- and 6-month-old 5xFAD mice**

**FIGURE S6 Ibrutinib suppresses CDK5 phosphorylation in 3- and 6-month-old 5xFAD mice**

**FIGURE S7 Ibrutinib reduces COX-2 levels in the cortex and dentate gyrus in 3-month-old PS19 mice**

**FIGURE S8 Ibrutinib does not alter Tau5 levels in 3-month-old PS19 mice**

**FIGURE S9 Oral administration of ibrutinib improves long-term memory and spinogenesis in 3-month-old 5xFAD mice**

**FIGURE S10 The BTK-specific inhibitor CC-292 does not alter dendritic spinogenesis in primary hippocampal neurons**

**FIGURE S11 Ibrutinib enhances hippocampal p-PI3K levels in 3-month-old PS19 mice**

**2 | Supplementary tables**

**TABLE S1** Distribution analysis of ibrutinib in brain tissue

**TABLE S2** List of antibodies used in this study


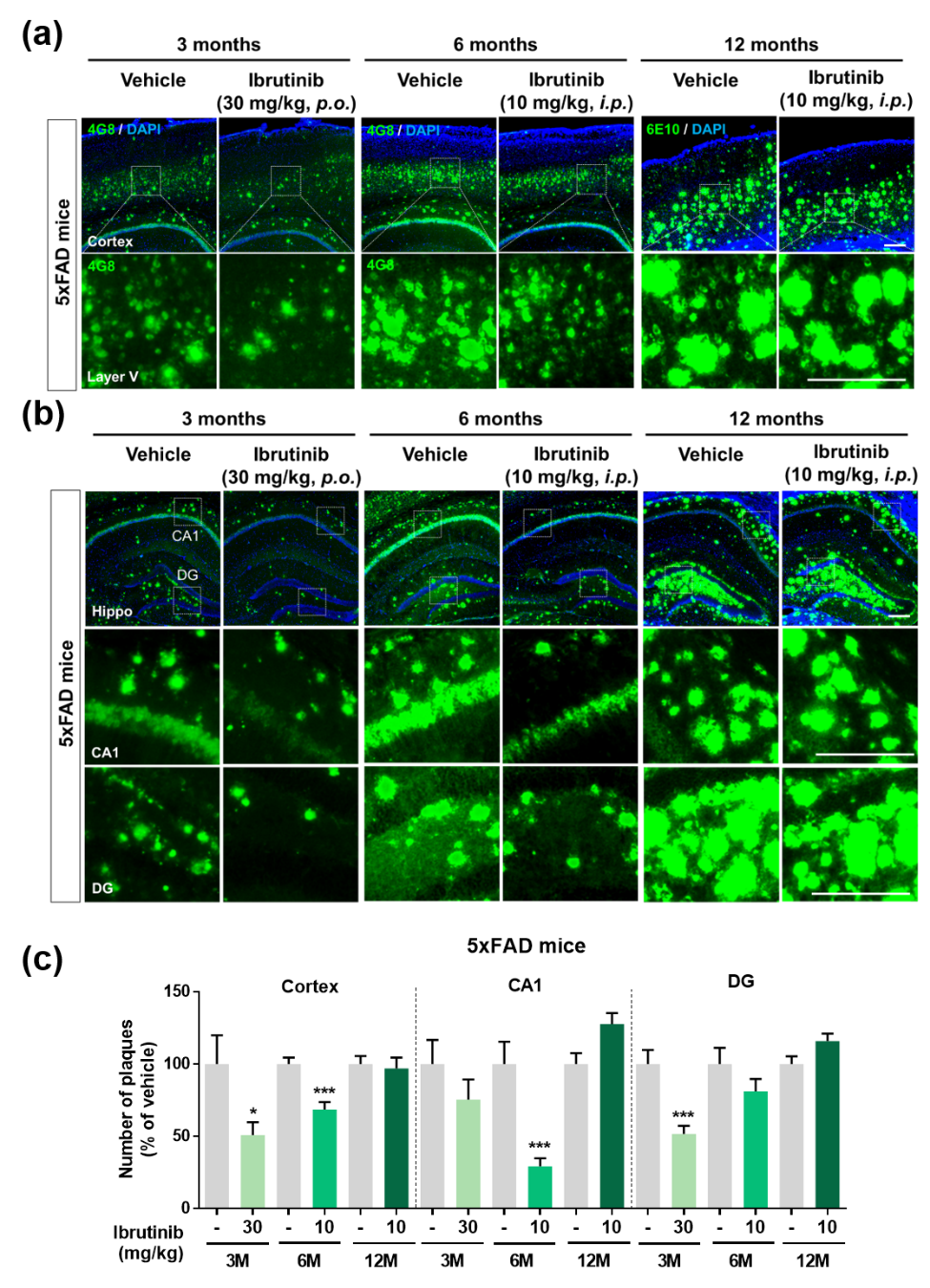


**FIGURE S1** Ibrutinib suppresses Aβ plaque levels in 3- and 6-month-old 5xFAD mice. (a and b) Ibrutinib (30 mg/kg, *p.o*.) or vehicle was administered to 3-month-old 5xFAD mice by injection daily for 30 consecutive days, and brain sections were immunostained with an anti-4G8 antibody. For 6- and 12-month-old 5xFAD mice, ibrutinib (10 mg/kg, *i.p*.) or vehicle was administered by injection daily for 14 consecutive days, and brain sections were immunostained with anti-4G8 or anti-6E10 antibodies. (c) Quantification of data from a and b (n = 4-5 mice/group). Scale bar = 100 μm (cortex, CA1, DG) and 200 μm (hippocampus). Data are presented as the mean ± SEM (*^*^p* < 0.05 and *^***^p* < 0.001 vs. vehicle).

**
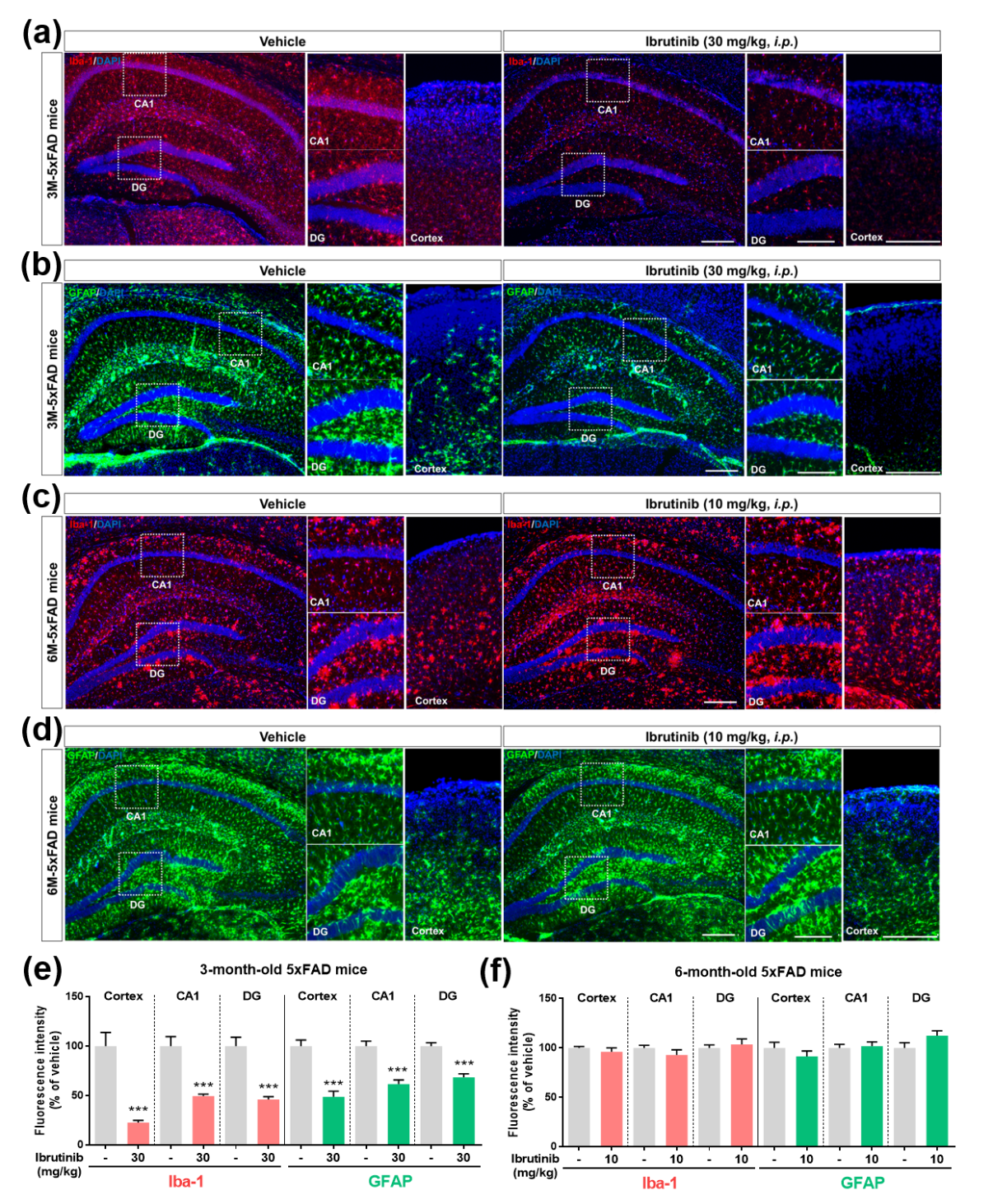
**

**FIGURE S2** Ibrutinib downregulates microglial and astrocyte activation in 3-month-old 5xFAD mice but not 6-month-old. (a-d) Ibrutinib at the indicated doses or vehicle was administered by injection (*i.p.*) daily for 14 consecutive days, and brain sections were immunostained with anti-Iba-1 and anti-GFAP antibodies. (e and f) Quantification of data from a and b and from c and d (3-month-old 5xFAD mice, n = 2/Iba-1 group; others, n = 4 mice/group). Scale bar = 100 μm (cortex, CA1, DG) and 200 μm (hippocampus). Data are presented as the mean ± SEM (*^***^p* < 0.001 vs. vehicle).

**
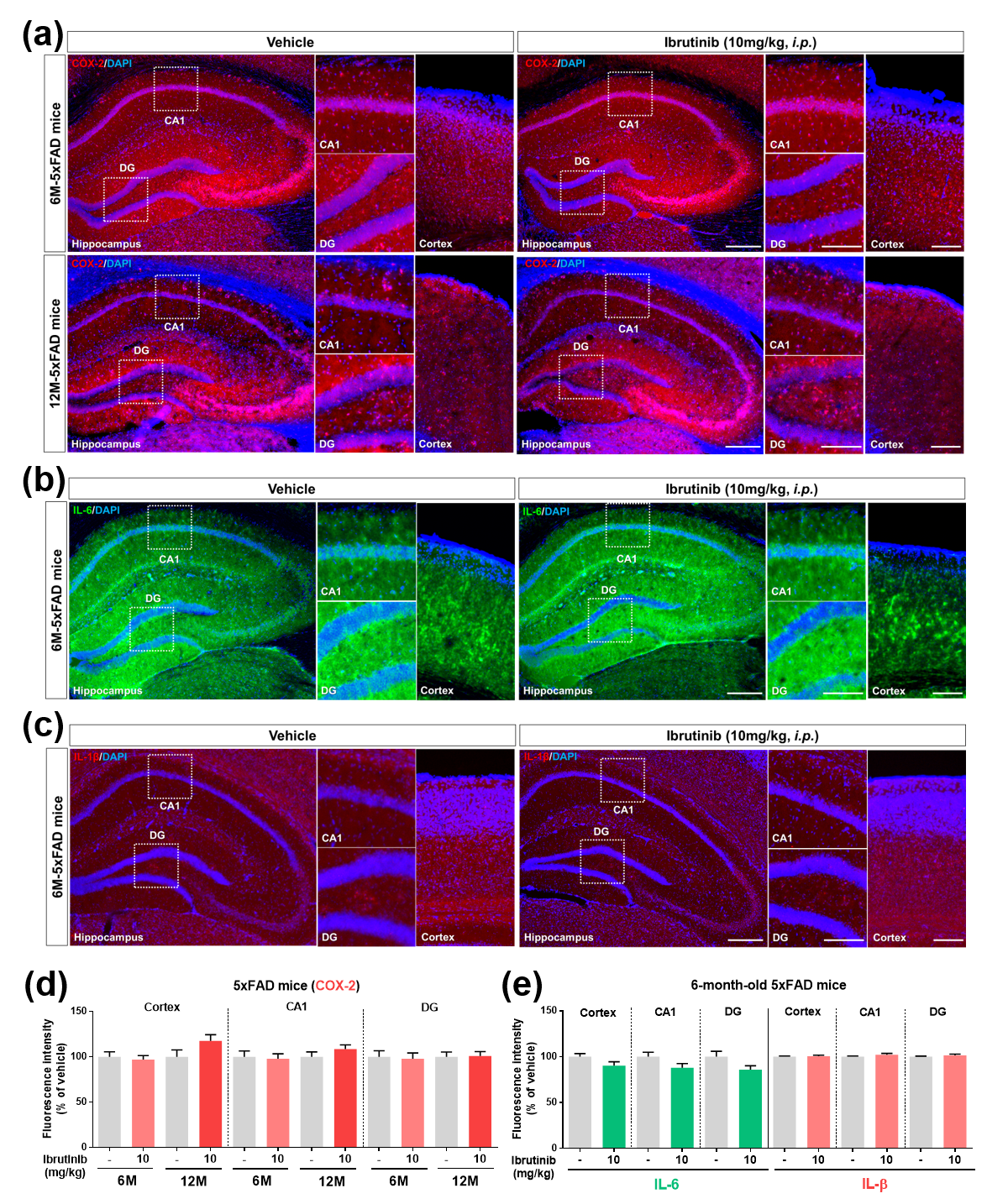
**

**FIGURE S3** Proinflammatory cytokine levels in 6- and 12-month-old 5xFAD mice are not altered by ibrutinib. (a-c) Ibrutinib or vehicle was administered by injection (*i.p.*) for 14 consecutive days, and brain sections were immunostained with anti-COX-2, anti-IL-6, and anti-IL-1β antibodies. (d and e) Quantification of data from a to c (n = 4-5 mice/group). Scale bar = 100 μm (cortex, CA1, DG) and 200 μm (hippocampus). Data are presented as the mean ± SEM.

**
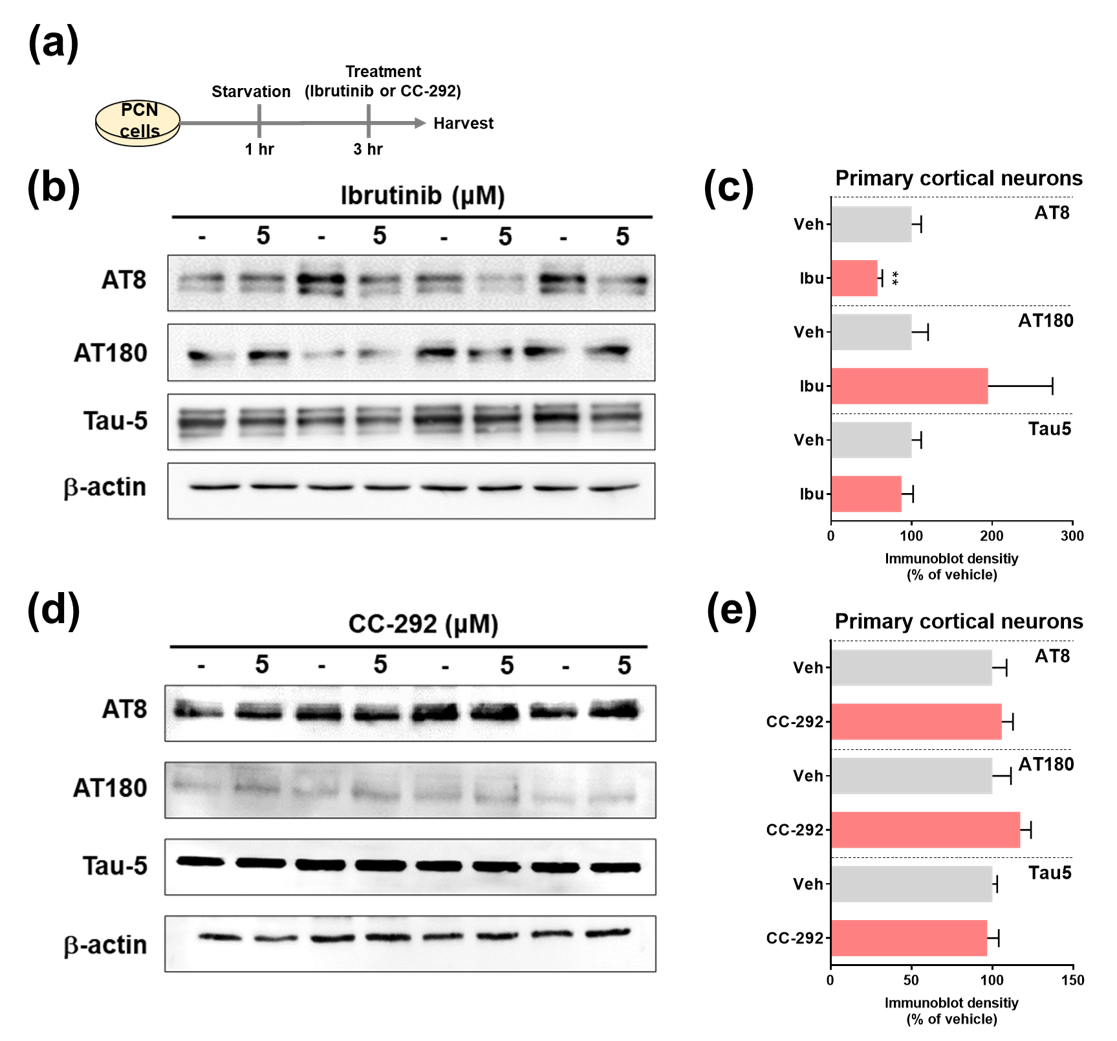
FIGURE S4** Tau phosphorylation is suppressed by ibrutinib but not by the BTK-specific inhibitor CC-292. (a) Primary cortical neurons were exposed to ibrutinib (5 μM), CC-292 (5 μM), or vehicle (1% DMSO) for 3 hr, followed by immunoblotting of the lysates with anti-AT8, AT180, and Tau-5 antibodies. The immunoblotting results for ibrutinib-treated **(b-c)** or CC-292-treated **(d-e)** primary cortical neurons were quantified and normalized to the vehicle group. Data are presented as the mean ± SEM (^**^*p* < 0.01 vs. vehicle). **PCN**: Primary cortical neurons

**
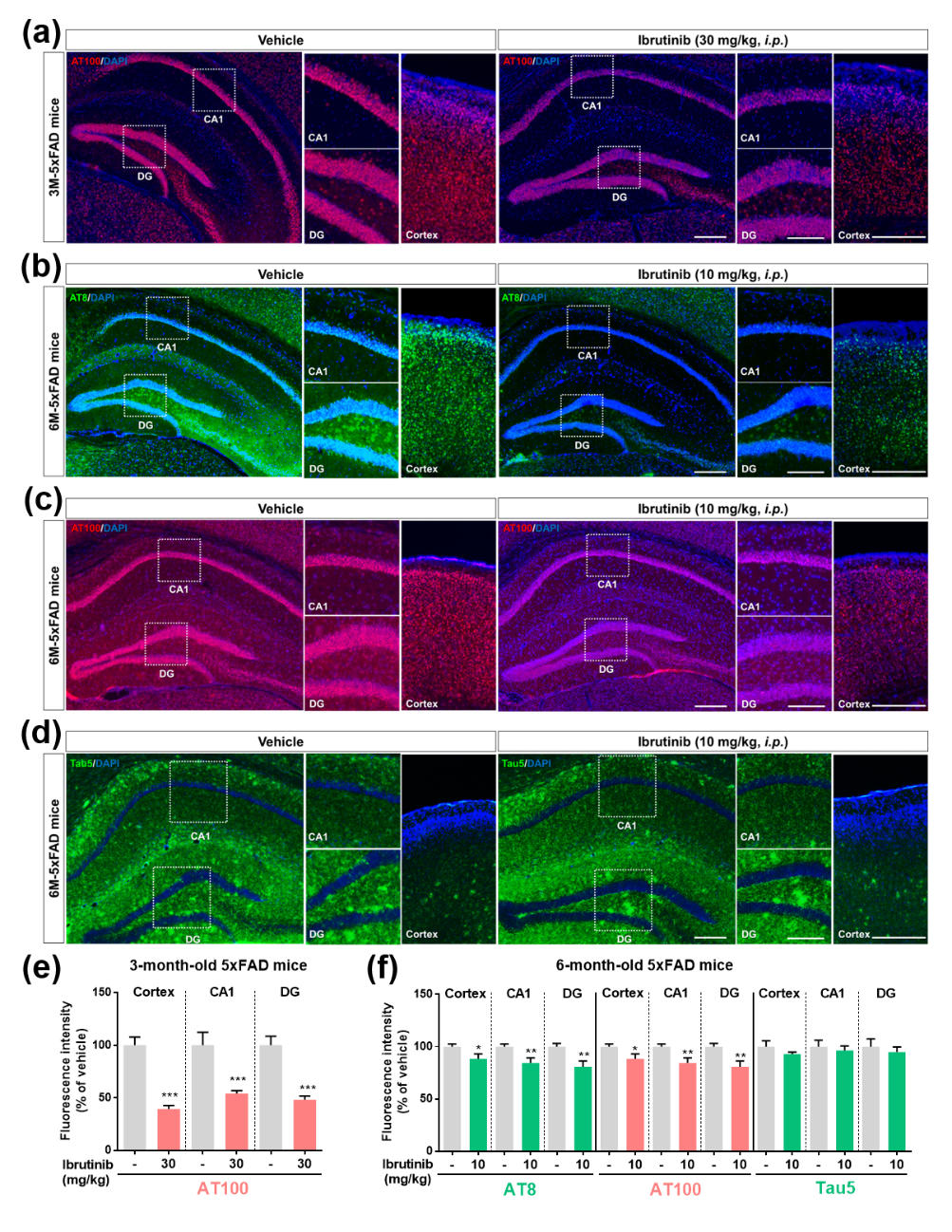
**

**FIGURE S5** Ibrutinib reduces tau phosphorylation in 3- and 6-month-old 5xFAD mice. (a) Ibrutinib (30 mg/kg, *i.p*.) or vehicle was administered to 3-month-old 5xFAD mice by injection daily for 14 consecutive days, and brain sections were immunostained with an anti-AT100 antibody. (b-d) Ibrutinib (10 mg/kg, *i.p*.) or vehicle was administered to 6-month-old 5xFAD mice by injection daily for 14 consecutive days, and brain sections were immunostained with anti-AT100, anti-AT8, and anti-Tau-5 antibodies. (e and f) Quantification of data from a to d (3-month-old 5xFAD mice, n = 2 mice/AT100 group; others, n = 4 mice/group). Scale bar = 100 μm (cortex, CA1, DG) and 200 μm (hippocampus). Data are presented as the mean ± SEM (*^*^p* < 0.05, *^**^p* < 0.01, and *^*^**^**^p* < 0.001 vs. vehicle).


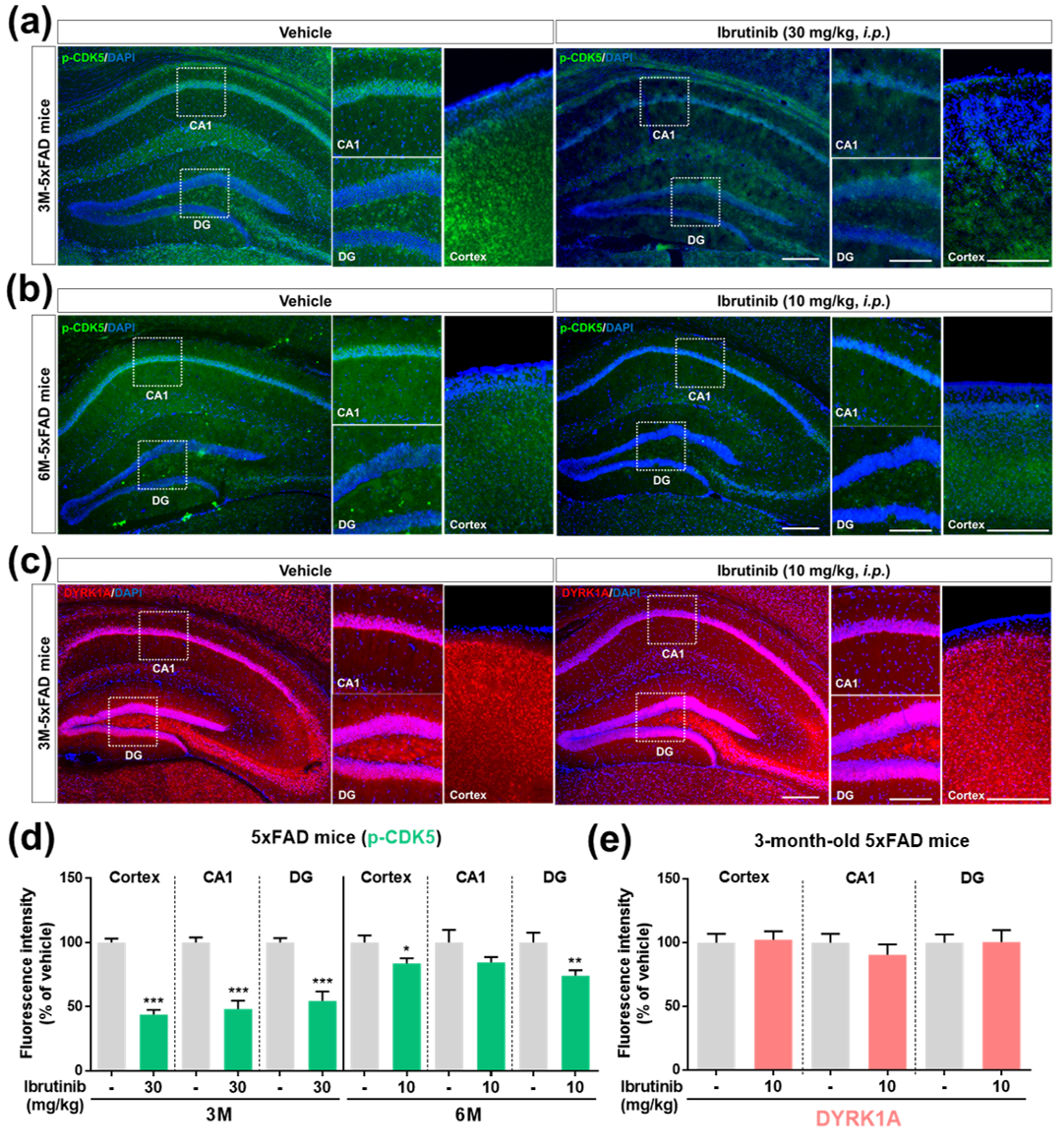


**FIGURE S6** Ibrutinib suppresses CDK5 phosphorylation in 3- and 6-month-old 5xFAD mice. (a and c) Ibrutinib (30 mg/kg, *i.p*.) or vehicle was administered to 3-month-old 5xFAD mice by injection daily for 14 consecutive days, and brain sections were immunostained with an anti-p-CDK5 antibody or anti-DYRK1A antibody. (b) Ibrutinib (10 mg/kg, *i.p*.) or vehicle was administered to 6-month-old 5xFAD mice by injection daily for 14 consecutive days, and brain sections were immunostained with an anti-p-CDK5 antibody. Scale bar = 100 μm (cortex, CA1, DG) and 200 μm (hippocampus). (d and e) Quantification of data from a to c (n = 3-5 mice/group). Data are presented as the mean ± SEM (*^*^p* < 0.05, *^**^p* < 0.01, and *^***^p* < 0.001 vs. vehicle).


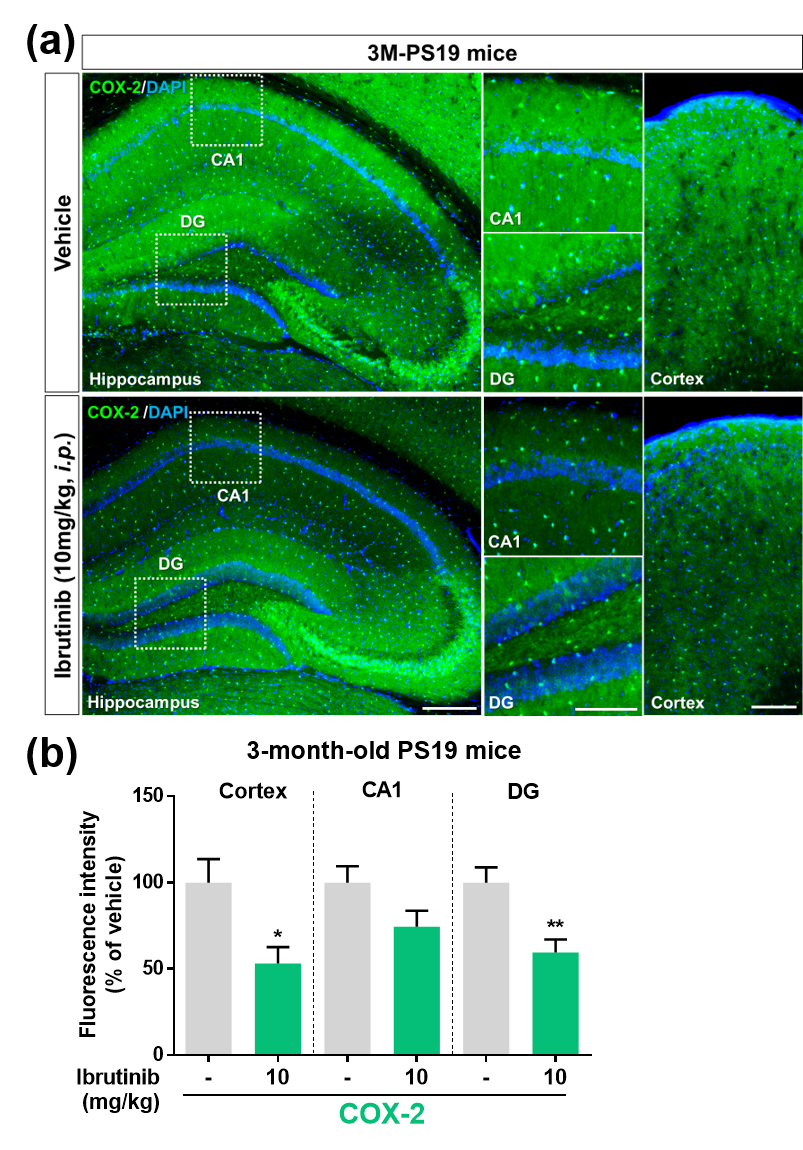


**FIGURE S7.** Ibrutinib reduces COX-2 levels in the cortex and dentate gyrus in 3-month-old PS19 mice. (a) Ibrutinib or vehicle was administered by injection (*i.p.*) daily for 14 consecutive days, and brain sections were immunostained with an anti-COX-2 antibody. (b) Quantification of data from a (n = 4 mice/group). Scale bar = 100 μm (cortex, CA1, DG) and 200 μm (hippocampus). Data are presented as the mean ± SEM (*^*^p* < 0.05 and *^**^p* < 0.01 vs. vehicle).


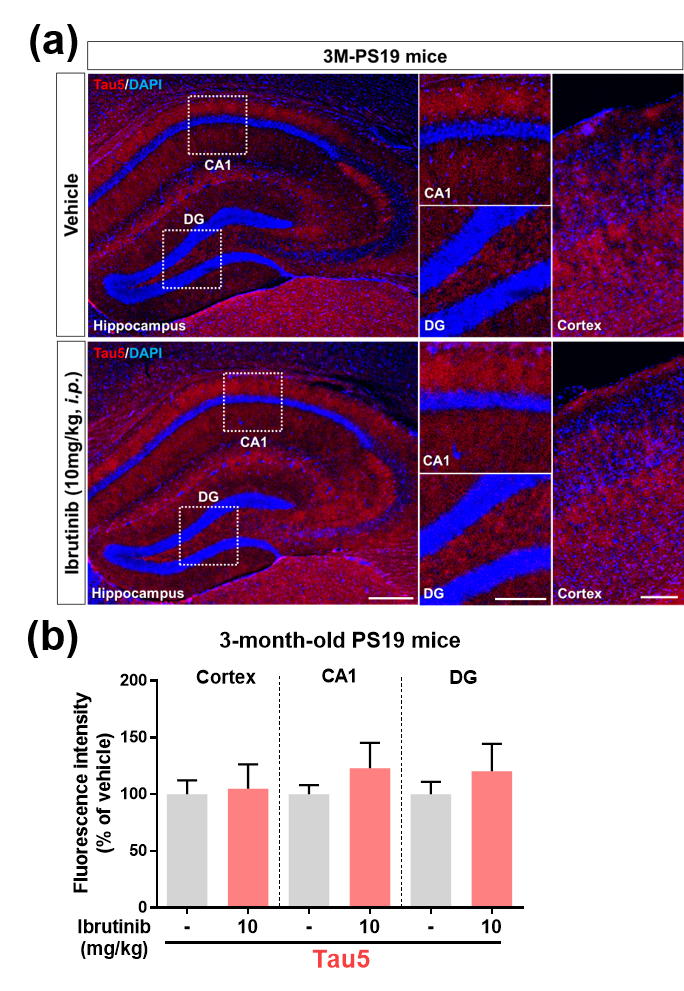


**FIGURE S8.** Ibrutinib does not alter Tau5 levels in 3-month-old PS19 mice. (a) Ibrutinib or vehicle was administered by injection (*i.p.*) daily for 14 consecutive days, and brain sections were immunostained with an anti-Tau-5 antibody. (b) Quantification of data from a (n = 4 mice/group). Scale bar = 100 μm (cortex, CA1, DG) and 200 μm (hippocampus). Data are presented as the mean ± SEM (*^*^p* < 0.05 and *^**^p* < 0.01 vs. vehicle).

**
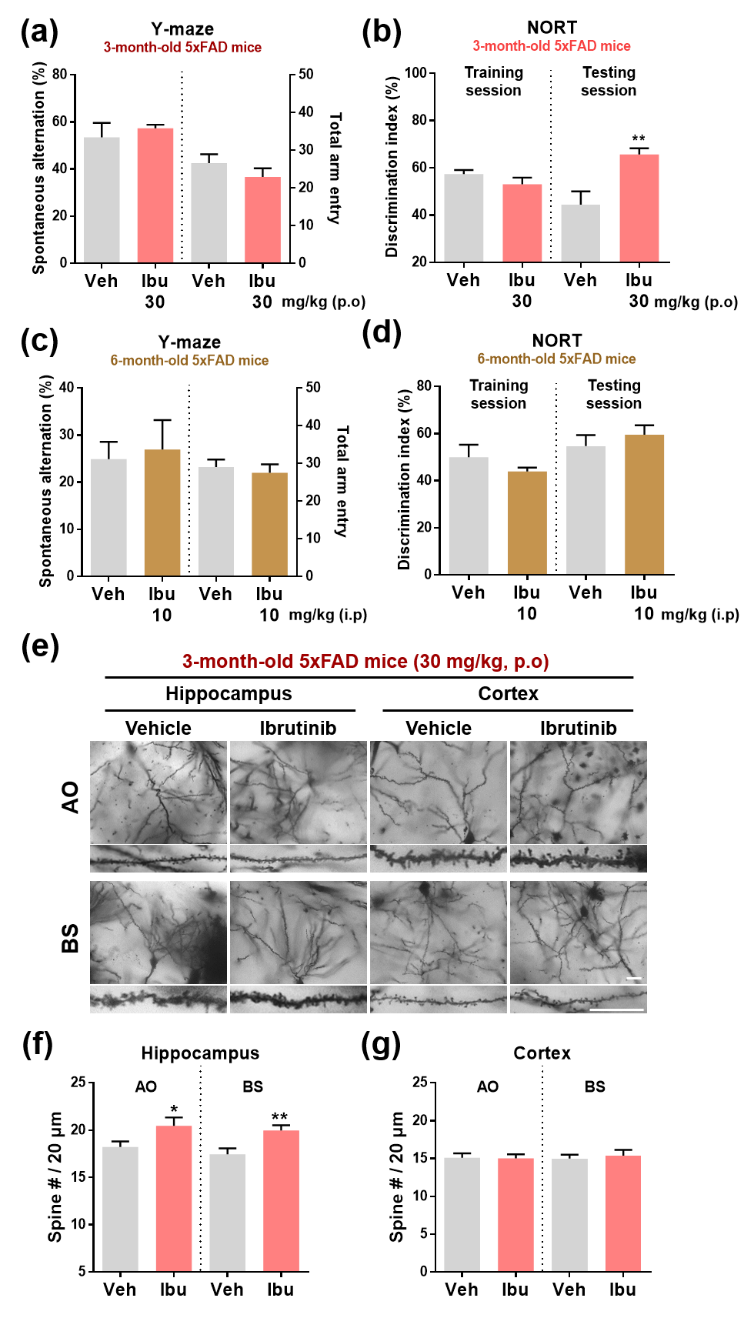
**

**FIGURE S9** Oral administration of ibrutinib improves long-term memory and spinogenesis in 3-month-old 5xFAD mice. (a-b) Ibrutinib (30 mg/kg, *p.o*.) or vehicle was administered orally to 3-month-old 5xFAD mice daily for 30 consecutive days, and behavior experiments were conducted. (c-d) Ibrutinib (10 mg/kg, *i.p.*) or vehicle was administered to 6-month-old 5xFAD mice by injection daily for 14 consecutive days, and behavior experiments were conducted. (e) Ibrutinib (30 mg/kg, *p.o*.) or vehicle was administered orally to 3-month-old 5xFAD mice daily for 30 consecutive days, and brain sections were subjected to Golgi staining. (f and g) Quantification of data from e (n = 4 mice/group). Scale bar = 20 μm. Data are presented as the mean ± SEM (*^*^p* < 0.05 and *^**^p* < 0.01 vs. vehicle).


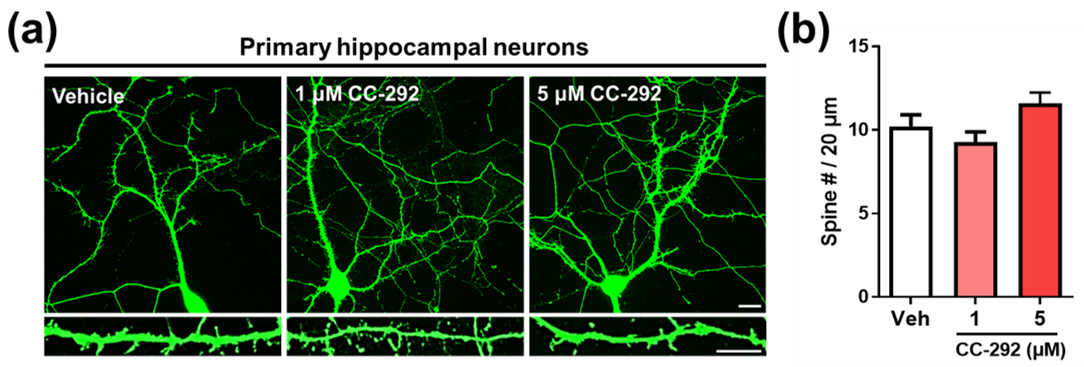


**FIGURE S10** The BTK-specific inhibitor CC-292 does not alter dendritic spinogenesis in primary hippocampal neurons. (a) Primary hippocampal neurons transfected with GFP plasmid DNA were exposed to CC-292 (1 or 5 μM) or vehicle (1% DMSO) for 24 hr. (b) Quantification of the number of dendritic spines per length (n = 17-20 dendrites derived from individual neurons). Scale bar = 20 μm. Data are presented as the mean ± SEM.


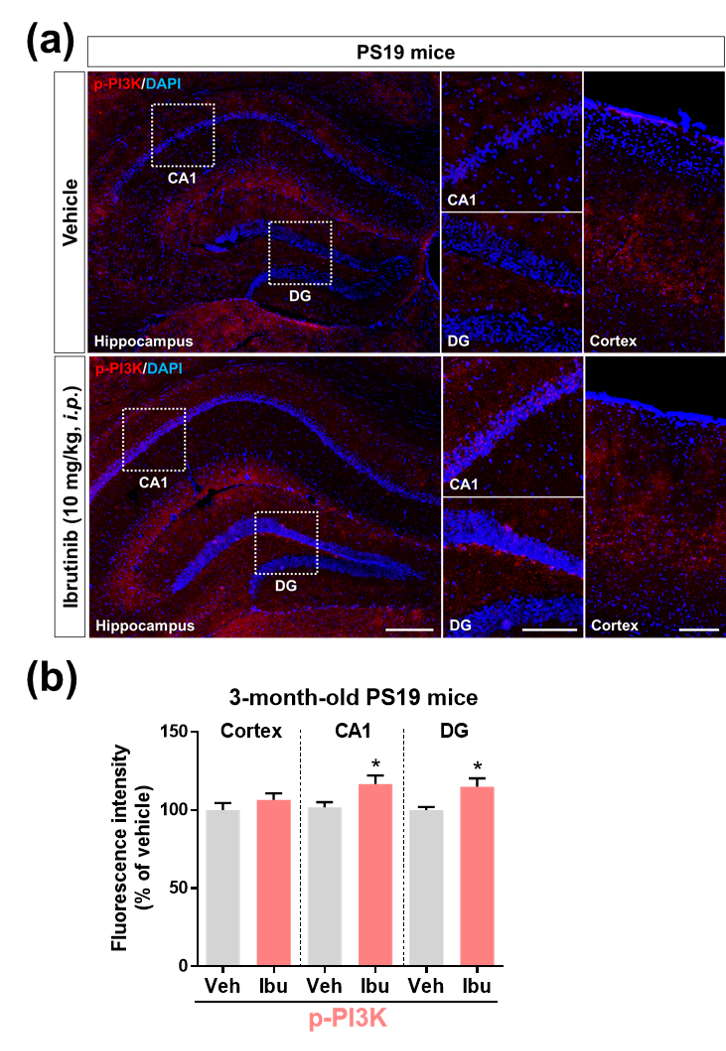


**FIGURE S11** Ibrutinib enhances hippocampal p-PI3K levels in 3-month-old PS19 mice. (a) Ibrutinib or vehicle was administered by injection (*i.p.*) daily for 14 consecutive days, and brain sections were immunostained with an anti-p-PI3K antibody. (b) Quantification of data from a (n = 4 mice/group). Scale bar = 100 μm (cortex, CA1, DG) and 200 μm (hippocampus). Data are presented as the mean ± SEM (*^*^p* < 0.05 vs. vehicle).

**TABLE S1** Distribution analysis of ibrutinib in brain tissue

| **Sample name** | **Ibrutinib / Brain tissue homogenate (ng/g)** |
| --- | --- |
| **Veh-1** | **35.935** |
| **Veh-2** | **24.725** |
| **Veh-3** | **BQL** |
| **Veh-4** | **BQL** |
| **Ibrutinib-1** | **96.503** |
| **Ibrutinib-2** | **143.664** |
| **Ibrutinib-3** | **84.699** |
| **Ibrutinib-4** | **163.166** |
|  | |

Wild type-mice were injected with ibrutinib (10 mg/kg, *i.p.*) or vehicle daily for 14 consecutive days. The brain was extracted, and the brain hemispheres were weighed and homogenized in 400 μL of PBS. The brain homogenates were analyzed by Agilent 1290 high-performance liquid chromatography (Agilent, USA) with a Kinetex C18 column and Triple Quad 5500 mass spectrometry (Applied Biosystems, USA). **BQL:** Below Quantifiable Limit

**TABLE S2** List of antibodies used in this study

| **Primary antibodies** | | | | | |
| --- | --- | --- | --- | --- | --- |
| **Antigen** | **Host species** | **Dilution** | **Manufacturer** | **Catalog no.** | **Analysis** |
| 4G8 | Mouse monoclonal | 1:500 | BioLegend | 800704 | IHC |
| 6E10 | Mouse monoclonal | 1:500 | BioLegend | 803002 | IHC |
| sAPPα | Rabbit polyclonal | 1:1000 | BioLegend | 813501 | WB |
| C1/6.1 | Mouse monoclonal | 1:1000 | Covance | SIG-39152 | WB |
| β-actin | Mouse monoclonal | 1:1000 | Santa Cruz | SC-47778 | WB |
| Iba-1 | Rabbit polyclonal | 1:500 | Wako | 019-19741 | IHC |
| GFAP | Rabbit polyclonal | 1:500 | Neuromics | RA22101 | IHC |
| IL-1β | Rabbit polyclonal | 1:100 | Abcam | AB9722 | IHC |
| COX-2 | Rabbit polyclonal | 1:200 | Abcam | AB15191 | IHC |
| IL-6 | Mouse monoclonal | 1:200 | Santa Cruz | SC-57315 | IHC |
| AT8 | Mouse monoclonal | 1:200 | Invitrogen | MN1020 | IHC, WB |
| AT100 | Mouse monoclonal | 1:200 | Invitrogen | MN1060 | IHC |
| AT180 | Mouse monoclonal | 1:500 | Invitrogen | MN1040 | WB |
| Tau5 | Mouse monoclonal | 1:200 (IHC)  1: 1000 (WB) | Invitrogen | AHB0042 | IHC, WB |
| p-CDK5 | Rabbit polyclonal | 1:200 | LSBio | LS-C354604 | IHC |
| DYRK1A | Rabbit polyclonal | 1:200 | Abcam | AB69811 | IHC |
| p-PI3K | Rabbit polyclonal | 1:200 | Cell Signaling | 4228 | IHC, ICC |
| GFP | Mouse monoclonal | 1:400 | Novus | NB600-597 | ICC |
|  |  |  |  |  |  |
| **Secondary antibodies** | | | | | |
| **Antibody** | | **Dilution** | **Manufacturer** | **Catalog no.** | **Analysis** |
| Goat anti-rabbit IgG, Alexa Fluor 555 | | 1:200 | Invitrogen | A21428 | IHC, ICC |
| Goat anti-rabbit IgG, Alexa Fluor 488 | | 1:200 | Invitrogen | A11008 | IHC |
| Goat anti-mouse IgG, Alexa Fluor 555 | | 1:200 | Invitrogen | A28180 | IHC, ICC |
| Goat anti-mouse IgG, Alexa Fluor 488 | | 1:200 | Invitrogen | A11001 | IHC |
| Goat anti-rabbit IgG, HRP conjugate | | 1:10000 | Enzo | ADI-SAB-300-J | WB |
| Goat anti-mouse IgG, HRP conjugate | | 1:10000 | Enzo | ADI-SAB-100-J | WB |
